# Role of B vitamins in modulating homocysteine and metabolic pathways linked to brain atrophy: Metabolomics insights from the VITACOG trial

**DOI:** 10.1002/alz.70521

**Published:** 2025-07-19

**Authors:** Tereza Kacerova, Abi G. Yates, Jiayi Dai, Dawn Shepherd, Elisabete Pires, Sebastian de Jel, Qingxia Gong, Eric Schiffer, Fredrik Jernerén, Thomas Olsen, Celeste A. De Jager Loots, Helga Refsum, A. David Smith, James S. O. McCullagh, Daniel C. Anthony, Fay Probert

**Affiliations:** ^1^ Department of Chemistry Chemistry Research Laboratory University of Oxford Oxford UK; ^2^ Department of Pharmacology University of Oxford Oxford UK; ^3^ numares AG Regensburg Germany; ^4^ Department of Pharmaceutical Biosciences Uppsala University Uppsala Sweden; ^5^ Department of Nutrition Institute of Basic Medical Sciences Faculty of Medicine, University of Oslo Oslo Norway; ^6^ The Ageing Epidemiology Research Unit School of Public Health Imperial College London Charing Cross Hospital London UK

**Keywords:** B vitamins, cognitive impairment, homocysteine, mass spectrometry, nuclear magnetic resonance, untargeted metabolomics

## Abstract

**INTRODUCTION:**

Elevated total homocysteine (tHcy) is a major predictor of brain atrophy, cognitive decline, and Alzheimer's disease (AD) progression. The VITACOG trial, a randomized, placebo‐controlled study in mild cognitive impairment (MCI), previously showed that B vitamin supplementation lowered tHcy, slowing brain atrophy and cognitive decline; however, the underlying mechanisms remained unclear.

**METHODS:**

We used untargeted, multi‐platform metabolomics, with nuclear magnetic resonance and liquid chromatography‐mass spectrometry to analyze serum samples from 89 B vitamin–treated and 84 placebo‐treated MCI participants over a 2 year follow‐up period.

**RESULTS:**

Multivariate modeling distinguished treated from placebo groups with 91.2 ± 1.8% accuracy. B vitamin supplementation induced significant metabolic reprogramming, lowering quinolinic acid, α‐ketoglutarate, α‐ketobutyrate, glucose, and glutamate.

**DISCUSSION:**

These findings reveal that B vitamins influence metabolic pathways beyond tHcy reduction, particularly the tricarboxylic acid cycle and glutamine–glutamate cycling, critical for brain energy homeostasis and neurotransmission. This metabolic signature supports B vitamin supplementation as a strategy for slowing MCI progression.

**Highlights:**

- Nuclear magnetic resonance and multi‐platform liquid chromatography tandem mass spectrometry metabolomics were performed on serum samples from 89 B vitamin–treated and 84 placebo participants in the VITACOG trial.
- Multi‐platform metabolomics revealed B vitamin–driven metabolic reprogramming, achieving 91% classification accuracy.
- B vitamin supplementation modulates key neuroprotective metabolic pathways.
- Regulation of energy metabolism and neurotransmission by B vitamins contributes to brain health in elderly individuals.
- B vitamins demonstrate potential as an adjunct therapy in mild cognitive impairment, potentially mitigating progression to Alzheimer's disease.

## Graphical abstract

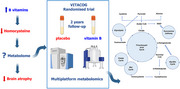

## 1 BACKGROUND

Individuals with mild cognitive impairment (MCI) exhibit an accelerated rate of brain atrophy, which correlates with an increased risk of developing Alzheimer's disease (AD).^1^, ^2^, ^3^ The VITACOG trial demonstrated that B vitamin supplementation reduces plasma total homocysteine (tHcy) and slows brain atrophy progression in older adults with MCI.^4^, ^5^ However, the mechanisms underlying this neuroprotective effect remain incompletely understood. Elevated tHcy is associated with greater cerebral atrophy and cognitive decline, but it is unclear whether tHcy directly contributes to neurodegeneration or reflects broader metabolic dysfunction, such as subclinical B vitamin insufficiency.^6^, ^7^, ^8^, ^9^ This distinction is important from both a mechanistic and clinical perspective, as it determines whether lowering tHcy levels alone can reduce brain atrophy in MCI, or whether tHcy is merely a surrogate biomarker for B vitamin status or other systemic alterations.

Hcy is a sulfur‐containing amino acid derived from methionine and metabolized via two B vitamin–dependent pathways: remethylation to methionine (requiring folate and vitamin B12) and trans‐sulphuration to cysteine (requiring vitamin B6).^10^ Deficiencies in these B vitamins have been reported to increase tHcy, which may exert direct neurotoxic effects through mechanisms including oxidative stress, excitotoxicity, and NAD⁺ depletion.^11^ Experimental models have demonstrated that Hcy inhibits neurite outgrowth in cerebellar Purkinje neurons^6^, ^7^ and induces apoptotic cell death in hippocampal neurons via DNA damage, activation of poly(ADP‐ribose) polymerase (PARP), and NAD⁺ depletion.^11^, ^12^ Notably, elevated tHcy can also result from factors beyond vitamin deficiency, such as renal dysfunction, genetic variation, and lifestyle.^13^, ^14^

While B vitamins are essential for Hcy metabolism, they also support a range of other biochemical pathways, including DNA methylation, neurotransmitter synthesis, and mitochondrial function.^15^, ^16^ The extent to which B vitamin supplementation modulates these broader metabolic networks in vivo, and whether such changes contribute to reduced brain atrophy, remains to be determined.

To investigate the metabolic effects of B vitamin supplementation and its relationship to Hcy lowering, we applied an untargeted metabolomics approach to serum samples from the VITACOG trial.^4^ This randomized controlled trial assessed the impact of high‐dose folic acid, vitamin B6, and vitamin B12 on plasma tHcy levels and cerebral atrophy in older adults.^4^, ^5^ Over 24 months, B vitamin supplementation significantly reduced tHcy and was associated with a 29.6% reduction in the rate of brain atrophy compared to placebo.^4^, ^17^ In the placebo group, baseline tHcy positively correlated with atrophy rate, whereas this association was absent in the treatment group.^4^ Although the trial also reported cognitive benefits in specific domains,^18^, ^19^ findings across studies remain inconsistent, highlighting the complexity of the relationships among tHcy metabolism, micronutrient status, and cognitive outcomes.^20^, ^21^, ^22^

Recent metabolomics studies have provided initial evidence linking B vitamin supplementation and tHcy reduction to metabolic alterations such as enhanced gluconeogenesis^23^ and decreased oxidative stress.^24^ However, the broader metabolic impact of B vitamins is poorly characterized. Untargeted metabolomics enables comprehensive profiling of the metabolome, allowing simultaneous assessment of diverse biochemical processes.^25^ This approach facilitates both pathway‐level interpretation and the identification of novel biomarkers, offering a robust framework to investigate the metabolic effects of B vitamins and their links to Hcy metabolism and cerebral atrophy.

Here, we used nuclear magnetic resonance (NMR) spectroscopy and mass spectrometry (MS) across two complementary platforms: reversed‐phase liquid chromatography MS (RPLC‐MS), targeting low‐ to medium‐polarity compounds, and anion‐exchange chromatography MS (AEC‐MS), which extends coverage to charged, polar metabolites relevant to B vitamin–dependent pathways.^26^ This combination—particularly the inclusion of AEC‐MS, uncommon in serum‐based studies, markedly improves the resolution of physicochemically diverse metabolites. The primary objective was to identify metabolites and pathways altered by B vitamin supplementation and the associated reduction in tHcy (see graphical abstract). By characterizing these changes within a functional biochemical framework, we aimed to elucidate the metabolic mechanisms underlying the neuroprotective effects of B vitamins in MCI and inform the optimization of future interventions.

## 2 METHODS

### 2.1 Participants and study design

The study used biobanked serum samples obtained from the clinical trial VITACOG, conducted in the Oxford, UK, area between April 2004 and November 2006.^4^ The patient inclusion criteria were defined by age > 70 years and diagnosis of amnestic or non‐amnestic MCI according to Petersen's criteria.^27^ Additionally, the exclusion criteria included a diagnosis of dementia or treatment with anti‐dementia drugs; active cancer; major stroke within past 3 months; treatment with methotrexate, anti‐cancer or anti‐epileptic drugs; or taking folic acid > 300 µg/d, pyridoxine > 3 mg/d, or vitamin B12 > 1.5 µg/d either per orally or any dose by injection.^4^ Individuals fulfilling entry criteria were randomized to either a treatment group or a placebo group. Randomization was conducted by an independent statistician using allocation concealment and minimization, accounting for age, sex, baseline Modified Telephone Interview for Cognitive Status (TICS‐M) score, and magnetic resonance imaging (MRI) consent. The treatment group received oral TrioBe Plus (Meda AB/Recip AB) containing 0.8 mg folic acid, 0.5 mg cyanocobalamin, and 20 mg pyridoxine HCl, or a placebo tablet.^4^ The treatment lasted 2 years. For a complete description of the study protocol, refer to the original study reports.^4^, ^18^ In this study, we focused on biologically compliant individuals, as described by Smith et al.^4^ Biological compliance was assessed based on changes in plasma B vitamin concentrations over a 2 year period, with specific cut‐off values used to categorize participants. In the treatment group, subjects were considered biologically compliant if they exhibited an increase in plasma folate of > 10 nmol/L and an increase in vitamin B12 of > 150 pmol/L from baseline to follow‐up. In the placebo group, compliance was defined as an increase of ≤ 10 nmol/L in folate and ≤ 150 pmol/L in vitamin B12.^4^ Participants who did not meet these criteria were classified as biologically non‐compliant, and were excluded from the present analysis.

### 2.2 Protocol approvals, patient consent

The VITACOG trial was conducted according to the principles described in the Declaration of Helsinki. The study was approved by a local National Health Service (NHS) research ethics committee (COREC 04/Q1604/100). Each involved individual provided written consent for their participation. The trial protocol and the Consolidated Standards of Reporting Trials (CONSORT) checklist are available in the original study by Smith et al.,^4^ while the updated CONSORT flowchart is available in the supporting information (see Figure S1). As a secondary analysis of biobanked samples from a completed clinical trial, this study adheres to Strengthening the Reporting of Observational Studies in Epidemiology (STROBE) guidelines, and the STROBE checklist for retrospective studies is available in the electronic clinical trial material.

**RESEARCH IN CONTEXT**

1. **Systematic review**: Total homocysteine (tHcy) is implicated in brain atrophy, cognitive decline, and increased Alzheimer's disease (AD) risk. Because folate, vitamin B12, and vitamin B6 serve as essential cofactors for tHcy metabolism, deficiencies in these nutrients have been linked to neurocognitive impairment. The VITACOG trial demonstrated that B vitamin supplementation lowers blood tHcy and slows cerebral atrophy; however, whether elevated tHcy actively drives neurodegeneration or serves as a biomarker of broader metabolic dysregulation remains debated.
2. **Interpretation**: Our findings demonstrate that B vitamin supplementation lowers tHcy while modulating central carbon metabolism, glutamine–glutamate cycling, and the kynurenine pathway. A multi‐platform metabolomics approach identified distinct metabolic signatures, achieving 91% accuracy in differentiating treated from placebo groups.
3. **Future directions**: Establishing whether B vitamins provide long‐term neuroprotection is essential, as expanding metabolomic analyses and longitudinal cognitive assessments will be key to elucidating their clinical significance and further supporting their role as a complementary therapy for mild cognitive impairment.

### 2.3 MRI acquisition and atrophy rate calculation

Volumetric cranial MRI scans were performed at baseline and after 2 years using a 1.5T MRI system (Siemens Sonata) at the Oxford Centre for Clinical Magnetic Resonance Research.^4^ The T1‐weighted 3D gradient echo (fast low angle shot [FLASH]) protocol used 1 mm isotropic voxels, a 19° flip angle, repetition time = 12 ms, and echo time = 5.65 ms, with 208 coronal slices per slab.^4^ Scans were repeated three times, averaged, and aligned. Brain atrophy rate was calculated using SIENA,^28^ an automated method, which estimates atrophy per year based on two scans taken 2 years apart. *Z* scores for the annual atrophy rate were calculated to classify participants with high‐quality MRI scans into two groups: progressors (positive *Z* scores, atrophy rate: 0.899–3.324) and stable individuals (negative *Z* scores, atrophy rate: −0.585 to 0.890). Additional voxelwise analyses of gray matter loss in this cohort, particularly in medial temporal and prefrontal regions, have been reported previously.^29^

### 2.4 NMR sample preparation and analytical methodology

The serum aliquots were thawed at 4°C and the samples were visually checked for any protein precipitation. All samples were processed according to a standardized protocol applied by Numares AG.^30^ The serum sample (405 µL) was diluted with 45 µL of D_2_O‐based buffer.^30^, ^31^ After NMR analysis, the processed NMR samples were recovered and stored at −80°C until the day of LC‐MS analysis.

All ^1^H NMR metabolomics experiments were performed at Numares AG, Regensburg, Germany, using a 600‐MHz Bruker AVIII ^1^H NMR spectrometer. For spectra acquisition, the zgpr30 pulse sequence with pre‐saturation to suppress the water signal was used, as previously described.^30^ The NMR spectra were manually phased, baseline corrected, and chemical shifts referenced to the lactate‐CH_3_ doublet signal (*δ* = 1.33 ppm) in Topspin 4.1.4. The processed spectra were then uploaded to the ACD/NMR processor academic edition 12.01 software (Advanced Chemistry Development, Inc.).^30^ The spectral regions between the 0.55 to 4.25 ppm and 5.20 to 8.50 ppm were divided into 0.02 ppm width bins, and the bins were sum‐normalized. The integrals of each bin were then exported for multivariate statistical analysis. The metabolite assignment was performed with reference to previous experiments, literature,^31^, ^32^, ^33^, ^34^ and the Human Metabolome Database.^35^

### 2.5 Serum sample preparation for LC‐MS analyses

The recovered NMR samples were thawed at 4°C and prepared for LC‐MS analysis. For RPLC‐MS analysis, metabolites from the recovered NMR samples were extracted by the addition of excess of acetonitrile (1:2.33 [v/v]). The samples were vortexed for 1 minute and centrifuged (16,000 × g; 15 minutes, 4°C). The supernatant was transferred into a clean microcentrifuge tube and centrifuged (16,000 × g; 15 minutes, 4°C). The supernatant was transferred into a total‐recovery MS autosampler vial.^36^

For AEC‐MS analysis, metabolites from the recovered NMR samples were extracted using a BuOH/MeOH mixture (1:4, v/v), added in excess to the NMR‐recovered serum at a ratio of 1:2.33 (v/v). The samples were vortexed for 1 minute and centrifuged (16,000 × g; 15 minute, 4°C). The supernatant was transferred into prewashed molecular weight cut‐off (MWCO) filtration columns (10 kDa MWCO filter; Amicon Ultra 0.5 mL centrifugal filters) and centrifuged (16,000 × g; 30 minute, 4°C). The filtrate was transferred into a total‐recovery MS autosampler vial.

A quality control (QC) sample for each extraction method was prepared by pooling 5 µL from each biological sample into a single total‐recovery MS autosampler vial. This QC sample was injected at the start of each LC‐MS sequence and after every 10 biological samples to evaluate data quality and ensure analytical consistency throughout the sequence. To further monitor overall system performance and retention time stability across platforms, a mixture of > 200 authentic metabolite standards was analyzed at both the beginning and end of the experimental sample sequence.

To exclude B vitamins and their direct metabolites from the results, we first identified these compounds by spiking serum samples (human serum derived from male AB blood; Merck) with selected B vitamin–related metabolites. A comprehensive list of the assessed compounds is provided in Tables S1–S3 in supporting information. This approach enabled the positive identification of these signals in the VITACOG study samples, ensuring their exclusion from statistical models and minimizing the risk of bias in the results.

### 2.6 AEC‐MS analysis

AEC‐MS analysis was performed using a Dionex ICS‐5000+ high‐pressure ion chromatography system equipped with a continuously regenerated trap column, Dionex ERS 500e suppressor, and AS11‐HC (2 × 250 mm, 4 µm) column, all from Dionex, coupled to a Q‐Exactive hybrid quadrupole‐Orbitrap MS via a HESI II probe (Thermo Fisher). The technical details of the AEC‐MS–based analysis have been previously described.^37^, ^38^

### 2.7 RPLC‐MS analysis

C18 RPLC‐MS analysis was performed using an Acquity Ultra Performance Liquid Chromatography (UPLC) system (Waters) operating under a gradient elution protocol, directly interfaced with a high‐resolution quadrupole time‐of‐flight mass spectrometer (Xevo G2‐XS QTOF, Waters). Sample injection was carried out using a 5 µL partial‐loop mode, with automated pre‐ and post‐injection needle wash steps included to minimize sample carryover. Full technical details of the RPLC‐MS analysis have been reported previously.^36^

### 2.8 Data processing

The untargeted metabolomics data (both for RPLC‐MS and AEC‐MS) were processed using Progenesis QI (Nonlinear Dynamics, Waters). The spectra were aligned and corrected for drift in the retention time. Data processing included peak picking, identification of natural abundance isotope peaks, detection of characteristic adducts (for all methods, [± H^+^] and [± H^+^—H_2_O] were applied), and metabolite identification using an in‐house database of standards. Metabolite identification was performed by matching multiple experimental parameters to the database of authentic standards, as previously described.^38^ The library comprised > 450 standards for AEC‐MS and 150 for RPLC‐MS. Level 1 identifications, as defined by the Metabolomics Standards Initiative (MSI), were assigned based on retention time (Rt error < 2 minutes), accurate mass (< 5 ppm for AEC‐MS and < 20 ppm for RPLC‐MS), and isotope and fragmentation pattern similarity (> 90%).^39^ All metabolites reported in Section 3 were further validated by co‐analyzing authentic standards alongside QC samples to ensure precise retention time alignment. The AEC‐MS method does not precisely resolve individual monosaccharides but reliably detects changes in C6 monosaccharides as a collective group. The most intense signal, represented by the overall chromatographic response, was primarily attributed to glucose due to its high abundance in serum. However, this signal likely also includes contributions from other hexose sugars present at lower abundances. Compound features and the identified metabolite list were filtered based on a coefficient of variance (%CV) threshold of < 30% in QC samples. Metabolites or compound features with a %CV > 30% in QC samples were excluded from further analysis. Ion intensity drift was corrected using the LOESS correction (*α* = 0.5) feature in MetaboDrift, with QC samples used to correct for within‐batch drift.^40^

### 2.9 Multivariate statistical analysis

The MetaboDrift corrected data were imported into R software 4.2.1 (R Foundation for Statistical Computing). Multivariate analysis was performed using in‐house R scripts and the ropls package.^41^ The NMR data were sum‐normalized and standardized using *Z* scores, while the LC‐MS data were sum‐normalized, log‐transformed, and standardized using *Z* scores. For NMR datasets, orthogonal partial least square discriminant analysis (OPLS‐DA) with 10‐fold cross‐validation, 100 repetitions, and permutation testing was performed, as previously described, to characterize the metabolomic differences between the B vitamin–treated individuals and placebo controls.^42^ For all models the reported accuracy, sensitivity, and specificity corresponds to the OPLS‐DA on test data (the model was trained blinded from this test set). The generated model ensemble was tested against 1000 randomly permuted models to determine its discriminatory capacity compared to random chance (as determined by a two‐sided Kolmogorov–Smirnov test, with a *p* value significance cut‐off of 0.05). the discriminatory metabolites and/or compound features were identified based on the average variable importance in projection (VIP) scores, representing the overall contribution of each variable to the OPLS‐DA model. For LC‐MS datasets and the models combining multiple metabolomic platforms, the assessment of metabolomic differences between treated individuals and placebo controls was conducted by a slightly altered version of the above‐described method. To prevent a drop in accuracy due to the input of a large number of variables, OPLS‐DA was combined with elastic net regularization. Elastic net regularization (*α* = 0.5) was applied, with cross‐validation to determine the optimal regularization parameter λ. Features were selected based on non‐zero coefficients from the regularized logistic regression model. Randomized null models were generated by shuffling classes to validate the robustness of the feature selection and classification performance. The OPLS‐DA was then performed as described above. For VIP scores, the overall score for a particular variable was determined as the frequency of selection divided by the average rank. Due to the large number of unidentified compound features in the LC‐MS datasets, complementary OPLS‐DA models were reconstructed using only identified metabolites. Metabolites with the highest VIP scores, particularly endogenous ones, were then subjected to univariate analysis.

### 2.10 Univariate statistical analysis

Univariate analysis was performed in R software 4.2.1 (R Foundation for Statistical Computing) using the ggplot2, ggpubr, effects, and dplyr packages. An unpaired two‐sample *t* test was applied for continuous variables, while the chi‐squared test was performed for categorical variables. A three‐way analysis of variance (ANOVA) was performed to assess the effects of treatment, atrophy progression, and time point on each metabolite, followed by pairwise comparisons using a Tukey post hoc test. Subsequently, a two‐way ANOVA was conducted to evaluate the effects of treatment, atrophy progression, and their interaction on each metabolite (follow‐up levels adjusted for baseline levels), followed by pairwise comparisons using a Tukey post hoc test. The significance cut‐off was set at *p* < 0.05 for all datasets; the false discovery rate (FDR) corrections (Benjamini–Hochberg) were applied as appropriate (if not indicated otherwise). Quantitative data are shown as Tukey boxplots (GraphPad Prism 10 software), with whiskers extending to the furthest values within 1.5 times the interquartile range (IQR). Outliers exceeding three times the IQR were identified and removed from the boxplot visualization to enhance interpretability. These extreme values were retained in the dataset for subsequent statistical analysis to assess their impact on the overall results.

A Pearson correlation analysis was performed using the corrplot R package to assess linear relationships among significantly altered metabolites, tHcy, folate, and vitamin B12 (all measured at follow‐up), as well as atrophy rate and cognitive test scores (Mini‐Mental State Examination [MMSE] and TICS‐M). A correlation matrix was calculated to assess the correlation coefficient (*α*) between pairs of variables.

### 2.11 Pathway analysis

Pathway topology enrichment analysis was performed using MetaboAnalyst 6.0,^43^ based on the KEGG metabolite library specific to *Homo sapiens* and incorporated global analysis of covariance (ANCOVA) enrichment analysis. The individual metabolites were allocated to metabolic pathways, and an estimate of metabolite importance within a metabolic network was determined based on a relative betweenness centrality algorithm. To ensure robustness, only metabolites with MSI Level 1 identifications were included in the pathway analysis.^39^

## 3 RESULTS

### 3.1 Cohort characteristics

To investigate the effects of B vitamin–induced tHcy lowering on the serum metabolome in the VITACOG cohort, we conducted a stratified analysis following the criteria established by Smith et al.^4^ The demographic and clinical characteristics of biologically compliant individuals are summarized in Table 1, with no significant baseline differences observed between the placebo and treatment groups. Although baseline folate levels did not differ significantly between groups (*p* = 0.173), they exhibited considerable variability, with greater dispersion in the placebo group (31.3 ± 25.9 nmol/L) compared to the treatment group (24.4 ± 15.1 nmol/L). The Shapiro–Wilk normality test confirmed a right‐skewed distribution, driven by two high outliers (> 90 nmol/L). Sensitivity analyses excluding these extreme values demonstrated no significant impact on metabolomic outcomes. To further assess group comparability, we also examined markers of kidney function, including estimated glomerular filtration rate (eGFR), which showed no significant differences between groups at baseline and follow‐up (Figures S2, S3 in supporting information), supporting the absence of renal confounding. To control for further confounding factors, we evaluated correlations between metabolite abundance and a range of clinical and behavioral variables, including age, smoking status, alcohol intake, and nutritional supplement use, with no significant associations observed (all *R^2^* < 0.2, *p* > 0.3). Additionally, co‐administered pharmacotherapies showed no discernible effect, as OPLS‐DA score plots demonstrated an even distribution across medications without distinct clustering (Figures S4–S6 in supporting information). Given previous VITACOG findings indicating reduced treatment efficacy with aspirin,^4^ signals corresponding to salicylic acid and acetylsalicylic acid were excluded from the analysis (Tables S1–S3 in supporting information).

**TABLE 1:** Demographic characteristics of placebo controls and individuals receiving B vitamin treatment.

Characteristics	Placebo *N* = 84	Treatment *N* = 89	*p* value
Age (baseline)	76.4 ± 4.6	77.0 ± 5.1	0.471
Sex (% female)	54 (64%)	55 (62%)	0.735
Years of education	15.4 ± 3.9	14.1 ± 3.4	0.312
Body mass index, kg/m^2^	26.1 ± 4.2	25.8 ± 3.5	0.471
Systolic blood pressure, mmHg	147 ± 18	145 ± 23	0.394
Diastolic blood pressure, mmHg	80 ± 10	79 ± 11	0.503
TICS‐M (baseline)	24.95 ± 2.81	24.91 ± 2.76	0.921
TICS‐M (follow‐up)	26.83 ± 4.24	26.71 ± 5.03	0.871
MMSE (baseline)	28.26 ± 1.47	28.19 ± 1.82	0.699
MMSE (follow‐up)	27.69 ± 2.23	27.81 ± 2.20	0.723
Initial brain volume (mL)^a^	1373.5 ± 67.4	1383.1 ± 87.2	0.482
Use of CVDs drugs (anytime)	43 (51%)	56 (63%)	0.119
Use of CNS drugs (anytime)	27 (32%)	39 (44%)	0.114
Use of aspirin (anytime)	34 (40%)	35 (39%)	0.877
tHcy (baseline), µmol/L	11.49 ± 3.19	12.06 ± 3.56	0.885
tHcy (follow‐up), µmol/L	13.01 ± 3.92	8.77 ± 1.90	<0.0001
Folate (baseline), nmol/L	31.3 ± 25.9	24.4 ± 15.1	0.173
Folate (follow‐up), nmol/L	23.6 ± 16.2	95.7 ± 38.9	<0.0001
Δ Folate from baseline, nmol/L	−4.80 ± 13.88	70.53 ± 40.46	<0.0001
Vitamin B12 (baseline), pmol/L	346.9 ± 95.8	342.1 ± 134.5	0.933
Vitamin B12 (follow‐up), pmol/L	342.8 ± 99.6	749.1 ± 276.3	<0.0001
Δ Vitamin B12 from baseline, pmol/L	5.72 ± 68.82	399.17 ± 232.90	<0.0001
Atrophy rate^a^	2.46 ± 0.49	0.73 ± 0.66	0.007

*Note*: Chi‐squared tests and unpaired two‐sample *t* tests were used to calculate *p* values for categorical and continuous variables, respectively. Age, BMI, and systolic and diastolic blood pressure were recorded at baseline. Continuous variables are presented as mean ± standard deviation (SD). “Baseline” refers to pre‐intervention, while “follow‐up” denotes post‐treatment assessments (2 years).

^a^ MRI data for atrophy rate determination were available for 62 individuals in the placebo group and 65 in the treatment group.

Abbreviations: BMI, body mass index; CNS, central nervous system; CVD, cardiovascular disease; MMSE, Mini‐Mental State Examination; MRI, magnetic resonance imaging; tHcy, total homocysteine; TICS‐M, Modified Telephone Interview for Cognitive Status.

### 3.2 Polar metabolome discriminates between B vitamin–treated individuals and placebo controls

**FIGURE 1:**
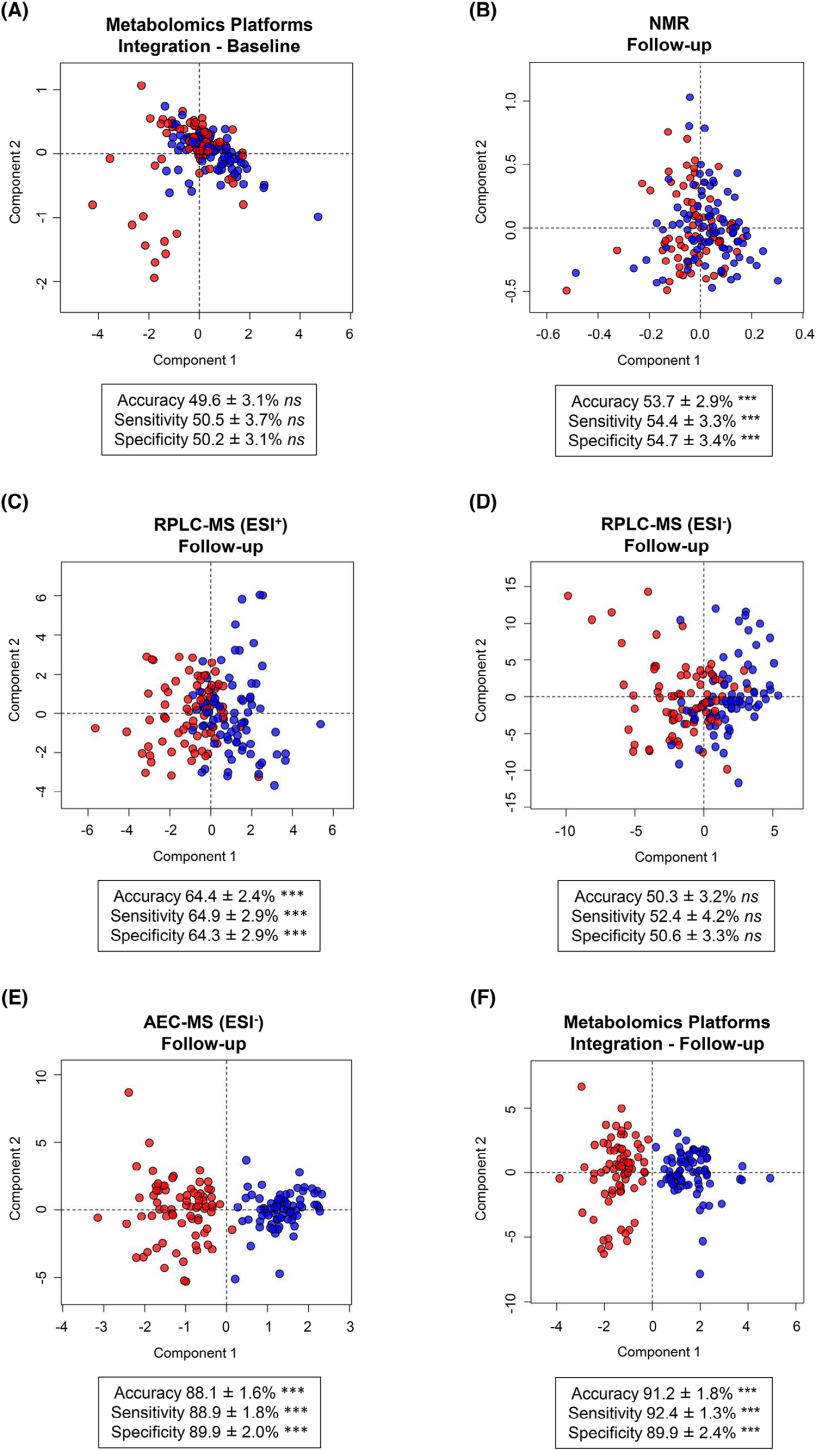
Orthogonal partial least squares discriminant analysis score plots illustrating the separation between biologically compliant placebo controls (red data points) and B vitamin–treated individuals (blue data points) based on serum metabolomic profiling. Results are presented for each individual metabolomic platform (follow‐up timepoint) and for the integrated dataset (baseline and follow‐up timepoints): (A) combination of all four platforms (baseline); (B) NMR (follow‐up); (C) RPLC‐MS (positive ionization mode; follow‐up); (D) RPLC‐MS (negative ionization mode; follow‐up); (E) AEC‐MS (negative ionization mode; follow‐up); (F) combination of all four platforms (follow‐up). “Baseline” refers to samples collected prior to any intervention, and “follow‐up” refers to samples collected after 2 years of B vitamin supplementation or placebo administration. Each model includes accuracy, sensitivity, and specificity (mean ± SD) derived from cross‐validation. Statistical significance (*p* > 0.05 ns; *p* < 0.001***) was assessed relative to a random class ensemble using the Kolmogorov–Smirnov test. AEC‐MS, anion‐exchange chromatography mass spectrometry; NMR, nuclear magnetic resonance; RPLC‐MS, reversed‐phase liquid chromatography mass spectrometry; SD, standard deviation.

To confirm the absence of baseline differences in the metabolome between experimental groups, we first constructed a multivariate model that integrated data from all analytical platforms and methods (Figure 1A). No statistically significant differences were observed at baseline, with the model achieving an accuracy of 49.6 ± 3.1%, which was not significantly different from the accuracy of a randomly permuted ensemble (*p* = 0.502). This established that any metabolomic differences observed post intervention are attributable to B vitamin supplementation rather than pre‐existing variability between the groups.

Having demonstrated a comparable baseline, we next evaluated the effects of B vitamins on the blood metabolome using untargeted metabolomic profiling of follow‐up serum samples from 89 B vitamin–treated individuals and 84 placebo controls. A multi‐platform approach combining NMR and LC‐MS was used, and multivariate OPLS‐DA models were constructed to characterize metabolic differences and capture the biochemical impact of B vitamin supplementation.

Results from the NMR analysis indicated limited ability to detect metabolic changes directly attributable to B vitamin supplementation, with model accuracy reaching 53.7 ± 2.9% (Figure 1B). While this accuracy was significantly above random (*p* = 2.4 × 10⁻^6^), the observed metabolic differences between treatment groups were subtle, likely reflecting the relatively small effect size of B vitamin supplementation on the high‐abundance metabolites detectable by NMR. The only metabolite identified from the VIP scores as statistically significant was alanine (*t* test *p* = 0.03), which was increased in B vitamin–treated individuals.

For LC‐MS, the discriminatory power varied according to the polarity of the detected metabolites. RPLC‐MS analysis in positive ionization mode demonstrated moderate discriminatory capacity, with an accuracy of 64.4 ± 2.4% (Figure 1C). In contrast, RPLC‐MS analysis in negative ionization mode revealed no significant metabolomic differences, with model accuracy comparable to a random model ensemble (50.3 ± 3.2%; *p* = 0.44; Figure 1D). In contrast, when the results from the AEC‐MS analysis (negative ionization mode) were modeled, an overall accuracy of 88.1 ± 1.6% was achieved. As AEC‐MS specifically characterizes highly polar and anionic compounds, this analysis highlighted the discriminatory potential of the polar metabolome in distinguishing B vitamin–treated individuals from placebo controls (Figure 1E). To evaluate the impact of data integration on group discrimination, we first assessed all binary combinations of the analytical platforms. These analyses demonstrated that the AEC‐MS dataset consistently provided the strongest discriminatory power, while the addition of other platforms yielded incremental gains in classification performance (Figure S7 in supporting information). We then combined data from all platforms to generate a single OPLS‐DA model, which achieved an accuracy of 91.2 ± 1.8% (Figure 1F), representing a modest yet statistically significant improvement over the AEC‐MS–based model alone (*p* = 1.32 × 10⁻^5^). Although AEC‐MS achieved the strongest classification, RPLC‐MS and NMR each contributed unique and complementary metabolic information, underscoring the value of a multi‐platform untargeted approach in discovery studies in which affected pathways are not known a priori.

### 3.3 B vitamin supplementation alters central carbon metabolism

Next, we aimed to identify metabolites and compound features significantly altered in the treatment group. Univariate analysis, in conjunction with multivariate models (Tables S4–S5 in supporting information), was used to delineate metabolomic changes driven by the treatment effect from those associated with brain atrophy progression. Consistent with findings from the original VITACOG study (Table 1),^4^ B vitamin administration was associated with a significant reduction in the rate of brain atrophy. However, not all individuals receiving B vitamin treatment exhibited a reduced atrophy rate, while a subset of placebo‐treated individuals showed minimal atrophy progression over the 2 year follow‐up period. To account for the potential confounding effects of brain atrophy, the cohort was stratified into four subgroups based on treatment allocation and atrophy rate, using MRI data from individuals with available scans (*N* = 127): B vitamin responders (*N* = 51); B vitamin non‐responders (*N* = 14); stable individuals in the placebo group (*N* = 31), representing metabolomic changes attributable to natural aging with MCI; and placebo individuals with worsening brain atrophy (*N* = 31), reflecting metabolomic changes associated with atrophy progression. Both baseline and follow‐up samples were included for all subgroups to account for temporal effects.

A three‐way ANOVA (Figure S8 and Tables S6–S8 in supporting information), incorporating treatment allocation (treatment vs. placebo), brain atrophy progression (stable vs. progressing), and timepoint (baseline vs. follow‐up), identified treatment allocation as the primary factor influencing the majority of metabolites. The AEC‐MS method identified statistically significant differences in the abundance of several exogenously derived compounds between groups, including mannitol, arabitol, and sorbitol, as well as saccharides less commonly observed in human blood, such as mannose and ribulose (Table S6). These results suggest alterations in metabolites associated with energy and sugar metabolism. To specifically assess the effects of B vitamins on the intrinsic metabolome, subsequent analyses primarily focused on endogenous metabolites. Several metabolites identified through the three‐way ANOVA exhibited significant treatment effects, while showing no significant associations with atrophy progression, timepoint effects, or their interactions, including: α‐ketoglutaric acid (treatment effect *p* < 0.0001 [FDR *p *= 0.002], Figure S8A), succinic acid (treatment effect *p* = 0.002 [FDR *p *= 0.03], Figure S8C), malic acid (treatment effect *p* = 0.0001 [FDR *p *= 0.008], Figure S8E), citramalic acid (treatment effect *p* = 0.0002 [FDR *p *= 0.008]), *N*‐acetylaspartate (treatment effect *p* = 0.0004 [FDR *p *= 0.01]), glutaric acid (treatment effect *p* = 0.0002 [FDR *p *= 0.009], Figure S8G), lactic acid (treatment effect *p* = 0.0004 [FDR *p *= 0.01], Figure S8H), pyruvic acid (treatment effect *p* = 0.001 [FDR *p *= 0.02], SI Figure 8I), and α‐ketobutyric acid (treatment effect *p* = 0.002 [FDR *p *= 0.03], Figure S8J).

Glutamic acid (treatment effect *p* < 0.0001 [FDR *p *= 0.0004]; atrophy effect *p* = 0.03 [FDR *p *= 0.21], Figure S8B), *N*‐acetylglutamate (treatment effect *p* = 0.0004 [FDR *p *= 0.01]; atrophy effect *p* = 0.03 [FDR *p *= 0.25], Figure S8F), and the glucose signal (treatment effect *p* = 0.0001 [*p *= 0.008]; atrophy effect *p* = 0.07 [*p = *0.39], Figure S8D) exhibited both significant treatment effects and moderate associations with atrophy progression. In contrast, quinolinic acid (treatment effect *p* = 0.02 [FDR *p *= 0.19]; atrophy effect *p* = 0.03 [FDR *p *= 0.24], Figure S8K) was one of the few metabolites showing a notable relationship with atrophy progression, with an effect size comparable to that observed for treatment (Tables S6–S8). These results suggest that quinolinic acid may be linked to both treatment response and atrophy progression, whereas other metabolites exhibited stronger associations with treatment effects.

As evidenced by the multivariate OPLS‐DA analysis, the NMR metabolome showed no clear discriminatory capacity between groups (Figure 1B). The three‐way ANOVA indicated that the only significantly altered metabolite was glutamine (Table S8), with a marginal treatment effect (*p* = 0.03 [FDR *p *= 0.38], Figure S8L) and no significant temporal or atrophy effects. Alanine, which ranked highly among the VIPs in the NMR‐based model, did not show a significant treatment effect (*p* = 0.13 [FDR *p* = 0.66]). Validation of NMR findings was limited by the LC‐MS methods, which exhibited poor retention and characterization of non‐derivatized amino acids, except for glutamate and aspartate detected via AEC‐MS.

**FIGURE 2:**
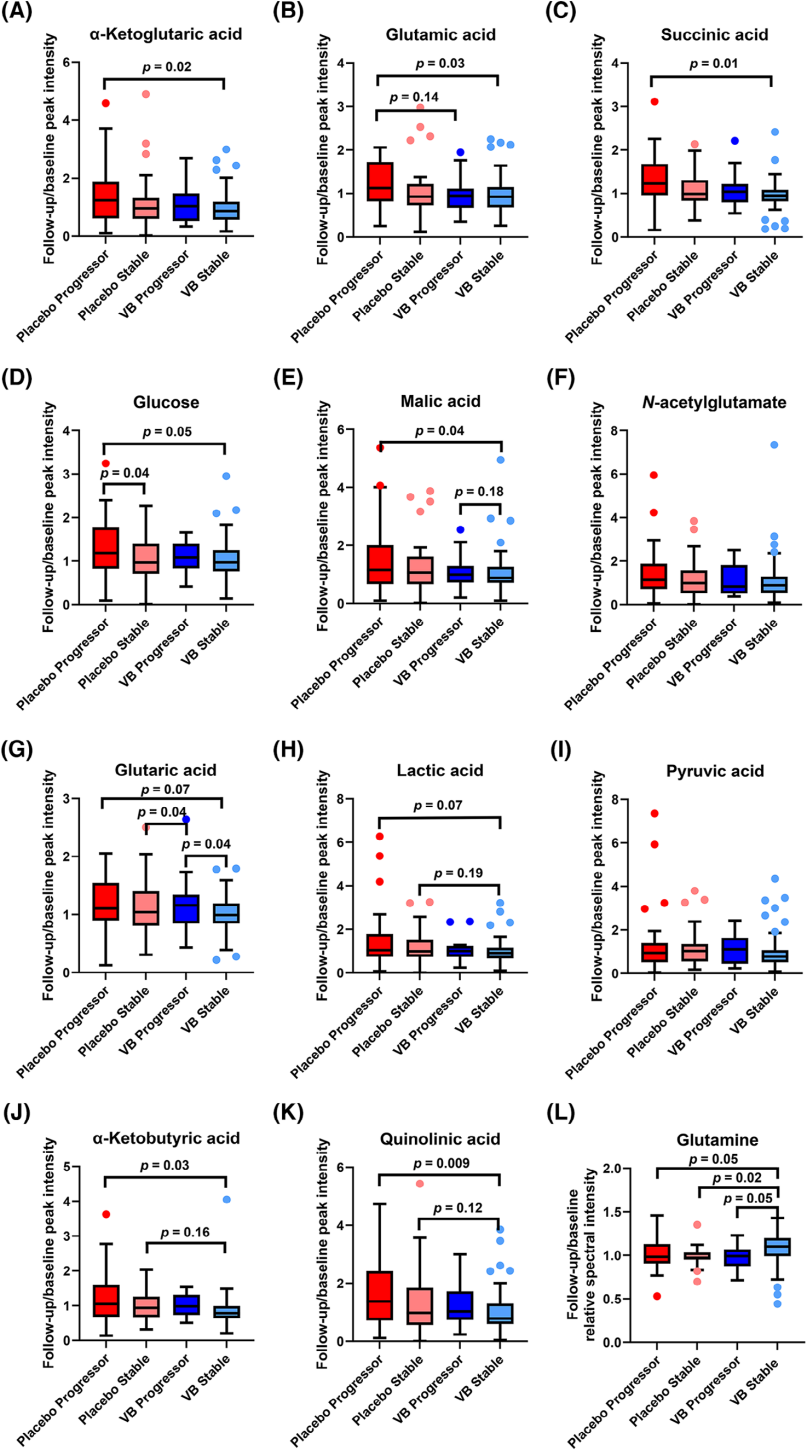
Results of two‐way analysis of variance comparing the relative serum abundance of individual metabolites across four groups, stratified by treatment status and brain atrophy progression as determined from magnetic resonance imaging over the 2 year follow‐up period: placebo progressors (dark red, *N* = 31), placebo stable (light red, *N* = 31), B vitamin progressors (non‐responders; dark blue, *N* = 14), and B vitamin stable (responders; light blue, *N* = 51). The figure presents follow‐up metabolite levels (adjusted for baseline) to enable interpretation of comparisons across treatment arms (placebo vs. B vitamins), progression status (progressors vs. stable), and within‐group treatment response (responders vs. non‐responders).Tukey post hoc analysis was performed, with results summarized in Table S9 in supporting information. Significant and marginal *p* values are displayed above the graphs; metabolites without displayed *p* values were not associated with any significant pairwise comparisons. A, α‐ketoglutaric acid (treatment *p *= 0.03; atrophy *p *= 0.32); (B) glutamic acid (treatment *p *= 0.06; atrophy *p *= 0.53); (C) succinic acid (treatment *p *= 0.03; atrophy *p *= 0.19); (D) glucose (treatment *p *= 0.12; atrophy *p *= 0.11); (E) malic acid (treatment *p *= 0.03; atrophy *p *= 0.48); (F) *N*‐acetylglutamate (treatment *p *= 0.20; atrophy *p *= 0.51); (G) glutaric acid (treatment *p *= 0.10; atrophy *p *= 0.36); (H) lactic acid (treatment *p *= 0.06; atrophy *p *= 0.41); (I) pyruvic acid (treatment *p *= 0.17; atrophy *p *= 0.66); (J) α‐ketobutyric (treatment *p *= 0.03; atrophy *p *= 0.17); (K) quinolinic acid (treatment *p *= 0.04; atrophy *p *= 0.15); (L) glutamine (treatment effect *p *= 0.19; atrophy effect *p *= 0.38). One outlier was excluded from the graphical representation to enhance data clarity. This exclusion did not significantly impact the results. VB, vitamin B.

To further account for potential temporal effects, a two‐way ANOVA was performed on follow‐up metabolite levels normalized to baseline, assessing the independent effects of treatment (B vitamins vs. placebo) and atrophy progression status (progressing vs. stable individuals). Consistent with the three‐way ANOVA results, treatment emerged as the primary factor driving metabolic changes. Tukey post hoc comparisons revealed that, for most metabolites, the most significant differences were observed between progressing individuals receiving placebo and those responding to B vitamin treatment, characterized by a stable rate of brain atrophy progression. These findings suggest that, while B vitamin administration exerts a primary influence on the metabolome, there may also be a modest additive effect associated with brain atrophy progression. The majority of metabolites, particularly intermediates of the tricarboxylic acid (TCA) cycle, glutamic acid, glucose, and quinolinic acid, exhibited moderate treatment effects, with some displaying varying degrees of association with atrophy progression (Figure 2, Table S9 in supporting information). Subtle differences were also observed within the B vitamin–treated group: for example, glutamine decreased (Figure 2L) and glutaric acid increased (Figure 2G) in non‐responders relative to responders, although these trends require validation in larger cohorts due to the limited number of non‐responders. Overall, metabolite levels decreased after B vitamin administration, especially in treatment responders, with the notable exception of glutamine (Figure 2L), which significantly increased in abundance in the treatment group.

Based on the three‐way ANOVA results, the following compounds were assessed (for details on the post hoc analysis, refer to Table S9): α‐ketoglutaric acid (treatment effect *p *= 0.03; atrophy effect *p *= 0.32; Figure 2A); glutamic acid (treatment effect *p *= 0.06; atrophy effect *p *= 0.53; Figure 2B); succinic acid (treatment effect *p *= 0.03; atrophy effect *p *= 0.19; Figure 2C); glucose (treatment effect *p *= 0.12; atrophy effect *p *= 0.11; Figure 2D); malic acid (treatment effect *p *= 0.03; atrophy effect *p *= 0.48; Figure 2E); *N*‐acetylglutamate (treatment effect *p *= 0.20; atrophy effect *p *= 0.51; Figure 2F); glutaric acid (treatment effect *p *= 0.10; atrophy effect *p *= 0.36; Figure 2G); lactic acid (treatment effect *p *= 0.06; atrophy effect *p *= 0.41; Figure 2H); pyruvic acid (treatment effect *p *= 0.17; atrophy effect *p *= 0.66; Figure 2I); α‐ketobutyric (treatment effect *p *= 0.03; atrophy effect *p *= 0.17; Figure 2J); quinolinic acid (treatment effect *p *= 0.04; atrophy effect *p *= 0.15; Figure 2K); glutamine (treatment effect *p *= 0.19; atrophy effect *p *= 0.38; Figure 2L).

### 3.4 Metabolomics shows limited ability to distinguish atrophy progression, despite correlation with polar metabolites

**FIGURE 3:**
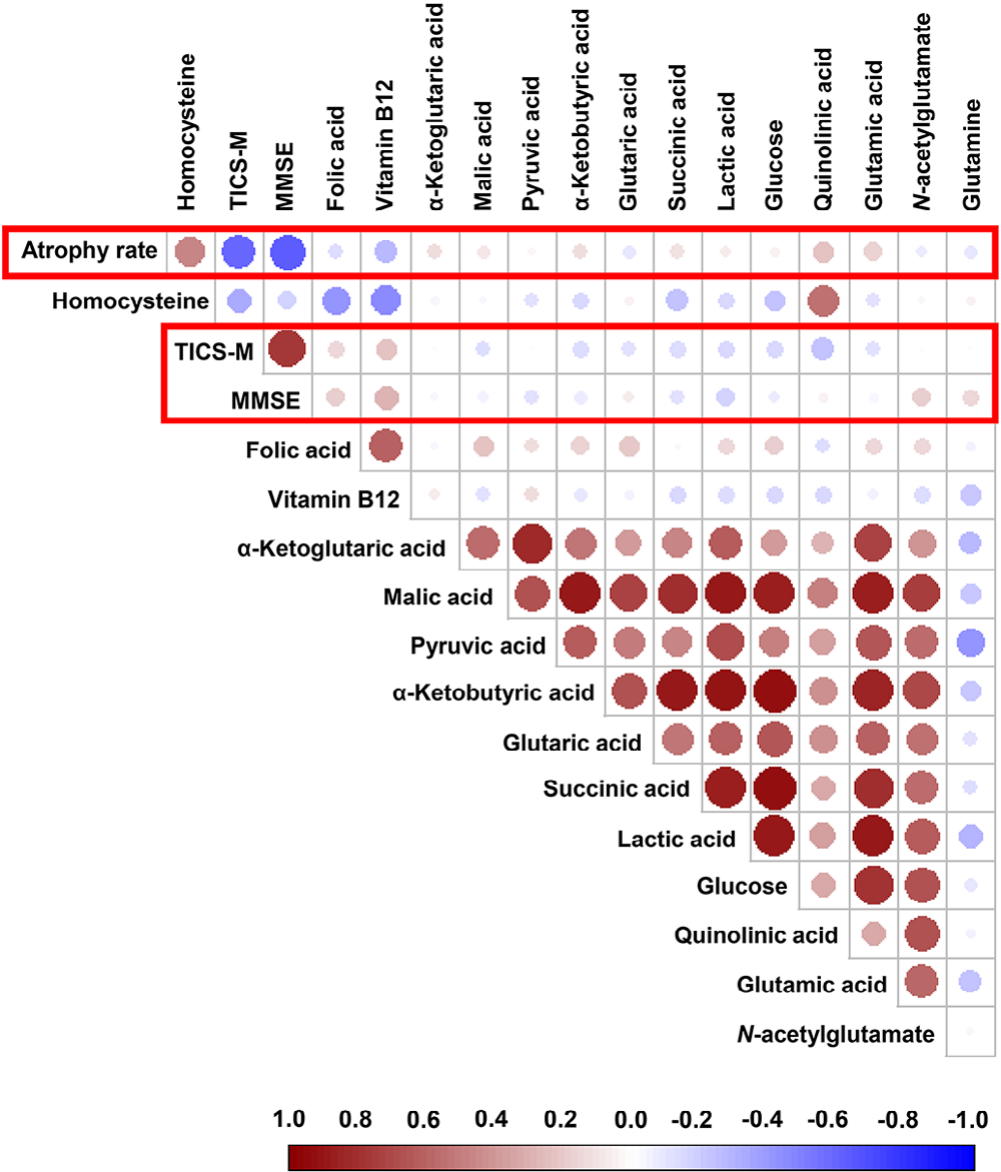
Pearson correlation analysis of follow‐up levels of significantly altered metabolites, homocysteine (total homocysteine), folate, and vitamin B12, along with atrophy rate and cognitive test scores (MMSE and TICS‐M, highlighted in the red box). Data from all compliant individuals with available magnetic resonance imaging scans at the follow‐up timepoint, receiving placebo and characterized by a fast rate of brain atrophy (“progressors”; *N* = 31). Color intensity reflects the magnitude of the Pearson correlation coefficient (α), with red indicating positive correlations and blue indicating negative correlations. MMSE, Mini‐Mental State Examination; TICS‐M, Modified Telephone Interview for Cognitive Status.

Building on the modest yet statistically significant effects of atrophy progression identified in the two‐way ANOVA post hoc analysis, further investigations were conducted to assess metabolic changes primarily associated with brain atrophy. OPLS‐DA models were constructed for placebo controls stratified by atrophy rate, analyzing stable and progressing individuals separately at both baseline (Figure S9A in supporting information) and follow‐up (Figure S9B). These models achieved similar accuracies of 64.3 ± 4.0% and 65.0 ± 4.4%, respectively, indicating only marginally detectable differences in the metabolome associated with brain atrophy. Furthermore, the results suggest that metabolic changes are present but remain relatively stable over the short follow‐up period, in contrast to the more dynamic alterations observed in response to B vitamin treatment. Although classification performance was modest, permutation testing confirmed the statistical significance of both baseline and follow‐up models (*p* < 0.0001), indicating that the metabolic differences between progressing and stable individuals were reproducible across timepoints. These findings align with univariate analyses, which also revealed metabolite‐level associations with brain atrophy. The comparable performance of models at both timepoints suggests that the metabolic phenotype associated with atrophy progression remains relatively stable over the 2 year follow‐up, potentially reflecting persistent, low‐grade metabolic dysregulation in this prodromal population. To further investigate the relationship between metabolite levels and atrophy progression, MRI data were used to quantify the rate of brain atrophy over the 2 year follow‐up period. This analysis focused on follow‐up samples from the subgroup of progressing individuals who received placebo (*N* = 31), thereby controlling for the potential confounding effects of treatment (Figure 3).

Pearson correlation analysis revealed a significant positive correlation between brain atrophy and tHcy (*α* = 0.47; Figure 3), consistent with previous reports.^4^ A strong negative correlation was observed between atrophy and cognitive scores at follow‐up (*α* = −0.58 for MMSE and *α* = −0.64 for TICS‐M; Figure 3), which was more pronounced in this subset than in the full cohort. This correlation was absent in the treatment group (Figure S10 in supporting information), potentially due to the treatment effect. Similarly, weak interactions between B vitamins and blood metabolites detected in the placebo group were not observed in the treatment group, except for glutaric acid, which positively correlated with both folate and vitamin B12 (Figure S10).

TCA cycle–related metabolites exhibited moderate correlations with brain atrophy progression (*α* ≈ 0.15). Stronger associations were observed for glutamic acid (*α* = 0.18) and quinolinic acid (*α* = 0.23). Most metabolites demonstrated negative correlations with Hcy, except quinolinic acid (*α* = 0.55), suggesting potential non‐linear or indirect pathway interactions between tHcy levels and atrophy progression. As expected, TCA cycle intermediates and their derivatives displayed strong intercorrelations (*α* > 0.5). Similarly, glutamine exhibited strong negative correlations with TCA cycle intermediates, particularly pyruvic acid (*α* = −0.40) and alpha‐ketoglutaric acid (*α* = −0.33). An inverse association between glutamic acid and glutamine was also identified (*α* = −0.24). Quinolinic acid demonstrated weaker correlations with TCA cycle metabolites, glutamic acid, and glucose (*α* < 0.30); however, these associations were stronger in the B vitamin responder group (*α* < 0.40; Fig. S10). Interestingly, most metabolites exhibited weak correlations with cognitive scores (MMSE and TICS‐M); however, in some cases, these correlations were unexpectedly stronger than those observed with atrophy. Notably, *N*‐acetyl glutamate (*α* = 0.20) and glutamine (*α* = 0.15) showed positive correlations with MMSE scores but not with atrophy (Figure 3). The observed discrepancies between correlations with MMSE, TICS‐M, and brain atrophy may reflect the limited sensitivity of standard cognitive assessments in detecting subtle metabolic alterations, particularly in individuals with MCI. These findings, derived from a relatively small sample size, should be interpreted as hypothesis generating and require validation in larger, independent cohorts. Notably, although several correlations reached statistical significance, effect sizes were generally modest (*α* ≈ 0.2–0.4), which is consistent with expectations in a clinically stable MCI population over a relatively short follow‐up period. This likely reflects the heterogeneous and low‐grade nature of peripheral metabolic changes in prodromal neurodegenerative states.

### 3.5 Metabolic pathway analysis revealed B vitamins effects on amino acid and central carbon metabolism

**FIGURE 4:**
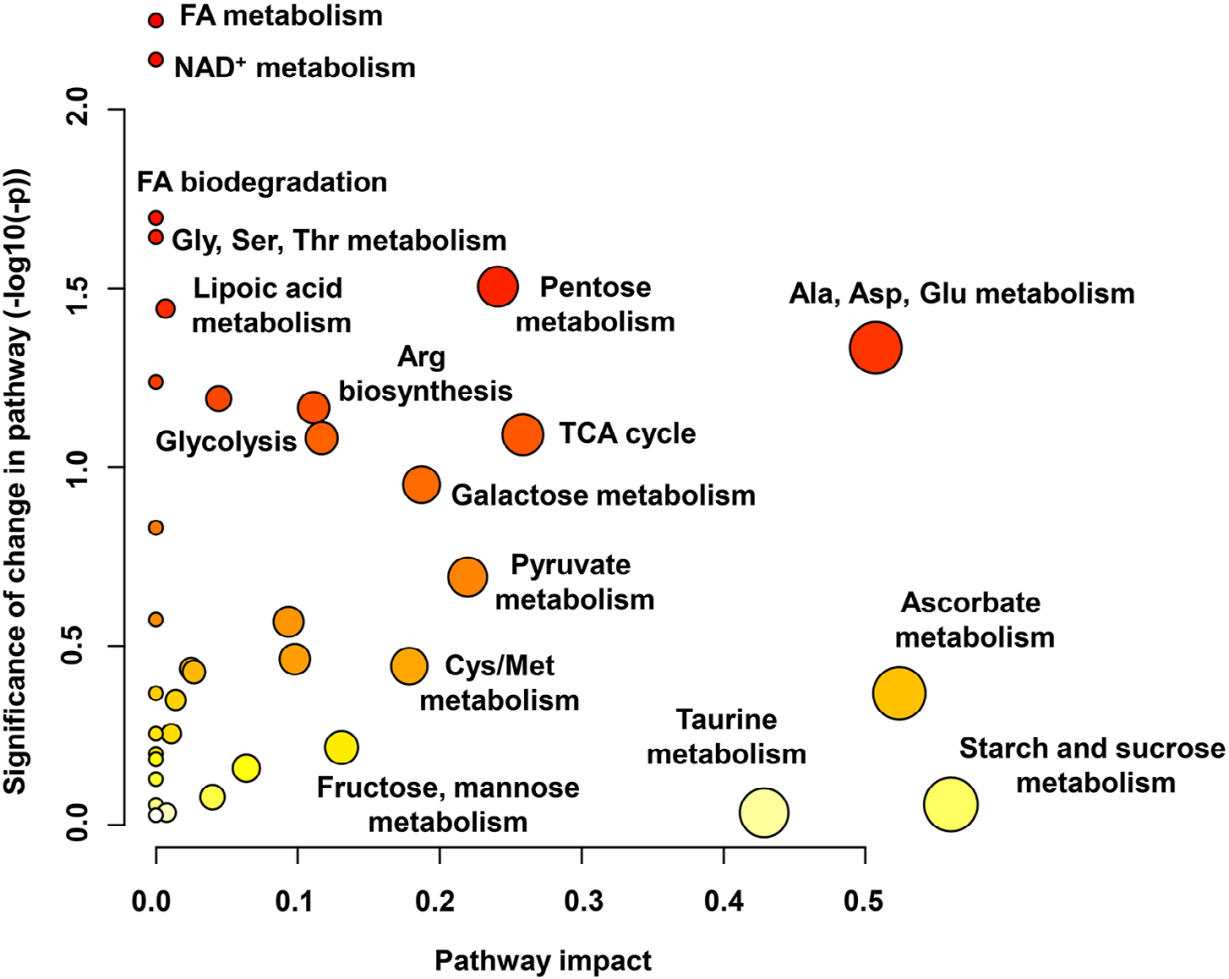
Metabolic pathway analysis of B vitamin–treated participants versus placebo controls. Individual metabolic pathways are represented as nodes, with node size indicating pathway impact (based on topological analysis) and color intensity (from white to red) reflecting pathway significance (based on enrichment analysis). A detailed description of the pathways, along with their associated significance and impact, is provided in Table S10 in supporting information. Fatty acids (FA) and amino acid metabolism (arginine, glycine, serine, threonine, alanine, aspartate, and glutamate) and central carbon metabolism (lipoic acid, NAD^+^, pyruvate metabolism, glycolysis, and the TCA cycle) were identified as the pathways most affected by B vitamin administration. Ala, alanine; Arg, arginine; Asp, aspartate; Cys, cysteine; Glu, glutamate; Gly, glycine; Met, methionine; Ser, serine; Thr, threonine; TCA, tricarboxylic acid.

To explore the possible impact and functional significance of B vitamin administration on metabolic processes, we conducted metabolic pathways analysis using enrichment and topographical methods with organism specificity for *Homo sapiens*. The pathways affected by B vitamin treatment (treatment vs. placebo) are illustrated in Figure 4.

**FIGURE 5:**
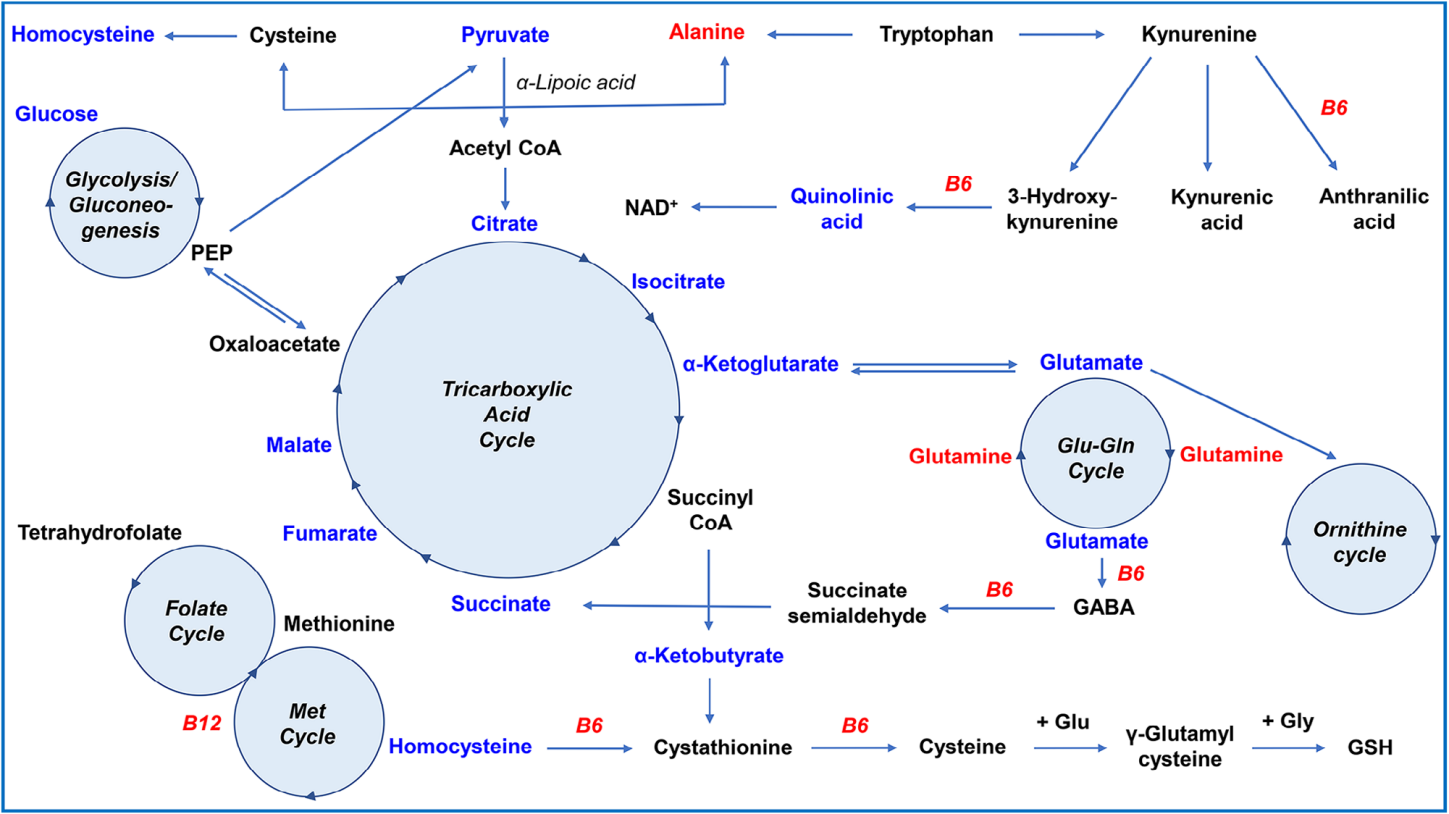
A simplified schematic of the metabolic networks related to and/or affected by B vitamin administration, as identified through multivariate, univariate, and pathway analyses. The primary pathways include glycolysis, the TCA cycle, glutamine/glutamate/γ‐aminobutyric acid (GABA) metabolism, the kynurenine pathway, and Hcy metabolism (including the folate and methionine cycles). Metabolites highlighted in blue exhibited decreased abundance after treatment, while those in red showed increased abundance. Metabolites shown in black were not detected by the analytical platforms used in this study. This schematic was created by the authors based on established metabolic pathways. Glu, glutamate; Gly, glycine; GSH, glutathione; Hcy, homocysteine.

The most significantly altered pathways (Figure 4, top; Table S10 in supporting information) included amino acid metabolism, particularly glycine, serine, and threonine, as well as arginine biosynthesis. Pathways linked to cysteine and methionine metabolism, both directly associated with Hcy metabolism, were identified by the analysis algorithm and exhibited a moderate impact. Although our methodology did not fully capture all aspects of fatty acid metabolism and biodegradation, we detected significant alterations in these pathways. Notably, lipoic acid and NAD⁺ metabolism, both critical for the conversion of pyruvate to acetyl‐CoA in the TCA cycle, were strongly affected (Figures 4, 5). The TCA cycle and pyruvate metabolism showed moderate but impactful changes, while glycolysis emerged as a key altered pathway, connecting to the TCA cycle via NAD⁺ and the conversion of phosphoenolpyruvate (PEP) to pyruvate (Figure 5). Saccharide‐involving pathways were also significantly altered, particularly pentose and galactose metabolism, with moderate changes observed in dietary saccharides such as starch and sucrose. Pathways involving glutamate and alanine metabolism showed alterations consistent with the three‐way ANOVA results. Notably, alanine and α‐ketoglutarate can be converted to pyruvate and glutamate via alanine aminotransferase (ALT), linking these pathways to the TCA cycle. Further pathways with high metabolic impact (Figure 4, right) included ascorbate metabolism, which, intriguingly, is not directly related to B‐vitamin metabolism. Taurine metabolism, a downstream product of the Hcy–cysteine pathway,^44^ did not exhibit significant metabolite‐level alterations but was identified as a key pathway with notable impact in the enrichment analysis. These results suggest that the primary pathways influenced by B vitamin administration are central carbon metabolism and amino acid metabolism, likely due to the role of B vitamins as cofactors for several enzymes involved in these processes. Figure 5 provides a schematic representation of the metabolic pathways predicted to be modulated by treatment, illustrating their interconnections with B vitamins and Hcy metabolism, particularly the methionine and folate cycles.

## 4 DISCUSSION

The VITACOG study previously reported that vitamins B6, B12, and folic acid significantly lowered tHcy and slowed cerebral atrophy in MCI.^4^, ^5^, ^8^, ^17^ While B6 and B12 act as cofactors and folate as a methyl donor in tHcy metabolism,^10^ the broader metabolic effects remain unclear. We conducted untargeted metabolomic profiling of serum samples using NMR and multi‐LC‐MS. B vitamin supplementation significantly decreased TCA cycle intermediates, glucose, glutamate, and quinolinic acid—metabolites interconnected with tHcy and folate metabolism. Although tHcy may be influenced by genetic, renal, or lifestyle factors, these were not associated with VITACOG outcomes.^4^ The randomized, placebo‐controlled design, absence of baseline imbalances, and minimal confounding in adjusted models support a causal role for B vitamin supplementation in driving the observed metabolic and neuroanatomical changes. Nonetheless, given the correlative nature of metabolomics, we cannot determine whether the identified metabolic shifts mediate the neuroprotective effects or represent downstream responses. Importantly, these metabolic effects were also reflected in brain atrophy patterns: quinolinic and glutamic acids correlated with atrophy progression, supporting a potential mechanistic link between B vitamin‐induced metabolic modulation and structural brain changes. While our multivariate models of atrophy progression demonstrated improved classification, predictive accuracy remained limited, underscoring the complexity of modeling individual trajectories in MCI. Nevertheless, to our knowledge, this is among the first studies to apply untargeted multi‐platform metabolomics in a clinically well‐characterized MCI cohort.

### 4.1 B vitamins lower serum glucose and TCA cycle intermediates

The primary metabolic changes in B vitamin–treated individuals included reduced serum glucose and altered TCA cycle intermediates. Impaired cerebral glucose metabolism, a hallmark of AD, results from defective insulin signaling and neuronal glucose accumulation.^45^ Additionally, AD is associated with deficiencies in glucose transporters, such as GLUT3 in neurons and GLUT1 in astrocytes, correlating with amyloid beta deposition.^46^, ^47^, ^48^ Our study noted a weak negative correlation between serum glucose and B vitamins, but a strong association between peripheral glucose and cerebral atrophy progression. Furthermore, glycolysis emerged as one of the most significantly altered pathways in enrichment analysis, indicating that a longitudinal increase in fasting plasma glucose is linked to higher brain tissue glucose concentrations in AD patients.^47^, ^48^ In the treatment group, we observed a significant decrease in lactate, a by‐product of anaerobic glycolysis that is often upregulated with mitochondrial dysfunction. Moreover, Hcy is implicated in both chronic inflammation and oxidative stress, where it promotes the formation of reactive oxygen species (ROS)^49^ that can impair insulin signaling and increase glucose concentration.^50^ Li et al. demonstrated that elevated Hcy may downregulate AMPKα/Sirt1 signaling, increasing oxidative stress.^50^ This suggests that B vitamin–induced reduction in tHcy levels may alleviate Hcy‐triggered oxidative stress and inflammation, potentially contributing to reduced blood glucose levels.

Correlation analysis identified a strong association between glucose and TCA cycle intermediates. Recent studies examining TCA cycle–related enzyme expression in neuronal cells have consistently reported decreased levels in AD brains, leading to TCA cycle inhibition and increased ROS production, which is suspected to accelerate neurodegeneration.^51^, ^52^, ^53^ However, peripheral alterations in TCA cycle enzymes or intermediates remain underexplored. As cofactors in synthesizing several TCA cycle substrates, B vitamins are expected to impact TCA cycle function. Folic acid and vitamin B12 catalyze the conversion of Hcy to methionine, producing nucleotides and amino acids like glycine or serine that are converted into pyruvate and then acetyl coenzyme A (CoA), entering the TCA cycle.^54^, ^55^ Similarly, vitamin B6 facilitates amino acid transamination, such as alanine, to form pyruvate, linking amino acid metabolism with central carbon metabolism and providing substrates for gluconeogenesis and the TCA cycle. We hypothesize that the observed decrease in TCA cycle intermediates after B vitamin supplementation reflects enhanced peripheral TCA cycle efficiency, due to improved cofactor availability. This may facilitate a more rapid conversion of intermediates into downstream products, such as NADH and FADH₂, which are crucial for ATP production. Consequently, this could prevent the accumulation of TCA cycle intermediates, explaining their reduced serum levels after B vitamin administration. Furthermore, the enhanced TCA cycle activity may also relate to improved mitochondrial function,^55^ suggesting that B vitamins counteract mitochondrial dysfunction in neurodegenerative conditions.

### 4.2 B vitamins indirectly affect glutamatergic and γ‐aminobutyric acid (GABA)ergic metabolism

A positive correlation was observed between glucose and glutamic acid, both of which significantly decreased after treatment. In the brain, glucose metabolism is crucial for energy production required for processes such as neurotransmitter release, reuptake, and recycling.^56^, ^57^ Impaired glucose metabolism and dysregulated glutamatergic signaling can lead to excessive glutamate release and excitotoxicity, contributing to neuronal damage and astrocyte dysfunction.^58^ The glutamate–glutamine cycle connects glutamate to the inhibitory neurotransmitter GABA. Among treatment responders, glutamine levels were significantly increased compared to placebo. An inverse correlation between glutamate and glutamine was observed in both groups, consistent with prior metabolomics findings.^59^ Vitamin B6, a cofactor for glutamate decarboxylase (converting glutamate to GABA) and GABA transaminase (converting GABA to succinate semialdehyde), may enhance these metabolic pathways,^60^ shifting equilibrium and contributing to reductions in glutamate, α‐ketoglutarate, and succinate. While the balance between excitatory (glutamate) and inhibitory (GABA) neurotransmission involves complex regulations beyond metabolite availability, the significant decreases in GABA‐associated metabolites after B vitamin treatment suggest an impact on neurotransmission.^61^, ^62^ Although GABA was not detected due to platform limitations, this pattern aligns with prior findings, which report contradictory GABA changes in AD—typically decreasing in the hippocampus and cortex, but increasing peripherally.^62^

### 4.3 B vitamins reduce quinolinic acid levels and modulate metabolic pathways linked to brain atrophy in MCI

Quinolinic acid levels were significantly reduced in individuals treated with B vitamins compared to placebo. Higher quinolinic acid levels were associated with greater brain atrophy, consistent with evidence linking elevated levels to poorer cognitive outcomes.^63^ However, reports on the role of quinolinic acid in dementia are mixed, with some studies reporting lower levels in AD despite associations with cognitive impairment.^64^, ^65^ As a downstream metabolite of tryptophan in the kynurenine pathway, quinolinic acid acts as an *N*‐methyl‐*D*‐aspartate (NMDA) receptor agonist, with overactivation linked to excitotoxicity and neuroinflammation in AD.^66^, ^67^ Hcy also activates NMDA receptors, sustaining their activity via phosphorylation signaling and promoting oxidative stress.^49^, ^68^ Simultaneous reductions in Hcy and quinolinic acid may reflect coordinated downregulation of neurotoxic signaling through shared excitatory pathways. Given their combined effects on NMDA receptor activation and neuroinflammation, these findings have broader implications for excitatory neurotransmission and neuronal survival, potentially offering new mechanistic insight into B vitamin–mediated neuroprotection. Dysregulation of the kynurenine pathway leads to quinolinic acid accumulation, observed, for example, in AD patient monocytes,^69^ which may disrupt neurotransmitter homeostasis and promote neuroinflammation via microglia activation and cytokine release.^66^, ^69^, ^70^ Additionally, quinolinic acid is strongly correlated with glucose, consistent with evidence that altered glucose metabolism influences the kynurenine pathway and may exacerbate neurodegeneration in AD and Parkinson's disease.^71^ Quinolinic acid was also positively correlated with TCA cycle intermediates, possibly owing to its conversion into NAD^+^, which is used by pyruvate dehydrogenase (PDH) to generate acetyl‐CoA for TCA cycle entry.^71^ This process also requires α‐lipoic acid, a PDH cofactor,^72^ identified as a key element in pathway enrichment analysis, suggesting an interplay between the kynurenine pathway and the TCA cycle.^70^ Notably, vitamin B6 supplementation reduced quinolinic acid levels despite its role as a cofactor in the kynurenine pathway. Although the atrophy progression model included both treatment arms and thus reflected a biologically heterogeneous cohort, multivariate modeling nonetheless identified consistent metabolic differences associated with brain atrophy, including elevated quinolinic acid levels. These findings were concordant with univariate and pathway‐level analyses, reinforcing their mechanistic relevance. In the context of B vitamin treatment, this may reflect complex regulation of pathway enzymes, such as kynurenine‐3‐monooxygenase, or negative feedback whereby elevated vitamin B6 levels reduce quinolinic acid production despite its cofactor role.^71^, ^73^

### 4.4 Limitations

Not all placebo‐treated individuals showed increased atrophy, nor did all B vitamin–treated individuals show reductions, indicating variable treatment responses over time. Stratifying participants by atrophy rate and treatment group allowed us to disentangle the effects of B vitamins, atrophy progression, and temporal metabolic changes. Although subgroup sizes were modest, this approach yielded valuable insights into treatment‐related metabolic responses. Baseline variability in dietary intake, microbiome composition, and serum folate levels may have contributed to metabolic heterogeneity and modulated responses. Although our multi‐platform approach provided broad metabolome coverage, it did not capture key lipid classes implicated in neurodegeneration, such as phospholipids and sphingolipids. Finally, pathway‐level interpretation is inherently constrained by the limited metabolic coverage and the structure of existing reference libraries; however, enriched pathways were cross‐referenced with prior literature to support biological plausibility.^74^ Given the hypothesis‐generating nature of untargeted metabolomics, further in vitro and in vivo studies are needed to validate B vitamin–induced metabolic changes and their relevance to neuroprotection.

### 4.5 Conclusion

This study provides a detailed characterization of serum metabolome changes in MCI after B vitamin supplementation and associated tHcy lowering. Untargeted NMR and LC‐MS profiling revealed shifts beyond tHcy, including modulation of central carbon metabolism and the kynurenine pathway—changes linked to brain atrophy. While exploratory, this is among the first multi‐platform metabolomics studies in a placebo‐controlled MCI trial. The findings offer a mechanistic framework for future work and support the potential of B vitamin supplementation as an adjunctive strategy for neurodegeneration.

## AUTHOR CONTRIBUTIONS

A. David Smith, Daniel C. Anthony, and Fay Probert conceived and designed the study. A. David Smith and Helga Refsum were responsible for study oversight and patient recruitment. Dawn Shepherd oversaw the biobank management. Tereza Kacerova, Abi G. Yates, and Jiayi Dai performed the experiments. Tereza Kacerova processed and analyzed the data. Elisabete Pires and Tereza Kacerova developed and optimized the sample processing methods. Fay Probert, Sebastian de Jel, Qingxia Gong, and Elisabete Pires oversaw the NMR analysis. James S. O. McCullagh oversaw the LC‐MS analysis. Fay Probert and James S. O. McCullagh provided supervision. Tereza Kacerova drafted the manuscript. Daniel C. Anthony, Fay Probert, James S. O. McCullagh, Fredrik Jernerén, Thomas Olsen, Helga Refsum, and A. David Smith provided critical feedback and guidance for experimental and analysis design. All authors provided input on the manuscript.

## CONFLICT OF INTEREST STATEMENT

A.D.S. and H.R. are named as inventors on two patents held by the University of Oxford on the use of B vitamins to treat cognitive disorders (US9364497 and US10966947). F.J. is named as an inventor on US10966947. These patents have been licensed to Elysium Health, NY. J.S.O.M. has a research contract and equipment loan from ThermoFisher Scientific, which manufactures IC‐MS systems. S. de J., Q. G., and E. S. are employees of Numares AG (Am Biopark 9, 93053 Regensburg‐Graß, Germany). All other authors report no conflicts of interest. Author disclosures are available in the supporting information.

## ETHICS STATEMENT

The trial, registered as VITACOG under the title “Homocysteine and B Vitamins in Cognitive Impairment” (ISRCTN 94410159), was conducted in accordance with the principles outlined in the Declaration of Helsinki. Ethical approval was granted by the local NHS research ethics committee (COREC 04/Q1604/100).

## CONSENT STATEMENT

Written informed consent was obtained from all participants prior to their enrolment in the study.

## Supporting information



Supporting Information

Supporting Information

## Data Availability

Anonymized data not published within this article will be made available by request from any qualified investigator.
