# Supplementary material for: Role of B vitamins in modulating homocysteine and metabolic pathways linked to brain atrophy: Metabolomics insights from the VITACOG trial
Source: Alzheimers Dement. 2025 Jul 19;21(7):e70521. doi: 10.1002/alz.70521 (PMC12276071; doi:10.1002/alz.70521)
Supplement: Supplementary file 2 — Supporting Information [file ALZ-21-e70521-s002.docx]

**Supplementary Information**

**Role of B vitamins in modulating homocysteine and metabolic pathways linked to brain atrophy: metabolomics insights from the VITACOG trial**

***Authors: Tereza Kacerova^1^, Abi G. Yates^1,2^, Jiayi Dai^1^, Dawn Shepherd^2^, Elisabete Pires^1^, Sebastian de Jel^3^, Qingxia Gong^3^, Eric Schiffer^3^, Fredrik Jernerén^4^, Thomas Olsen^5^,*** [***Celeste A. De Jager Loots***](https://pubmed.ncbi.nlm.nih.gov/?term=De+Jager+Loots+CA&cauthor_id=38297399)***^6^, Helga Refsum^2,5^, A. David Smith^2^, James S. O. McCullagh^1^*, Daniel C. Anthony^2^*, Fay Probert^1^***

****Corresponding authors:***

***James S.O. McCullagh*** [***james.mccullagh@chem.ox.ac.uk***](mailto:james.mccullagh@chem.ox.ac.uk)

***Daniel C. Anthony*** [***daniel.anthony@pharm.ox.ac.uk***](mailto:daniel.anthony@pharm.ox.ac.uk)

***Addresses:***

*^1^ Chemistry Research Laboratory, Department of Chemistry, University of Oxford, Mansfield Road, Oxford, OX1 3TA, UK*

*^2^ Department of Pharmacology, University of Oxford, Mansfield Road, Oxford, OX1 3QT, UK*

*^3^ numares AG, Am Biopark 9, Regensburg, 93053, Germany*

*^4^ Department of Pharmaceutical Biosciences, Uppsala University, Husargatan 3, Uppsala, 752 37, Sweden*

*^5^ Department of Nutrition, Institute of Basic Medical Sciences, Faculty of Medicine University of Oslo, Postboks 1046 Blindern, 0317 Oslo, Norway*

*6The Ageing Epidemiology Research Unit, School of Public Health, Imperial College London, Charing Cross*

*Hospital, St Dunstan's Road, London, W6 8RP, UK*

**CONSORT diagram**

**
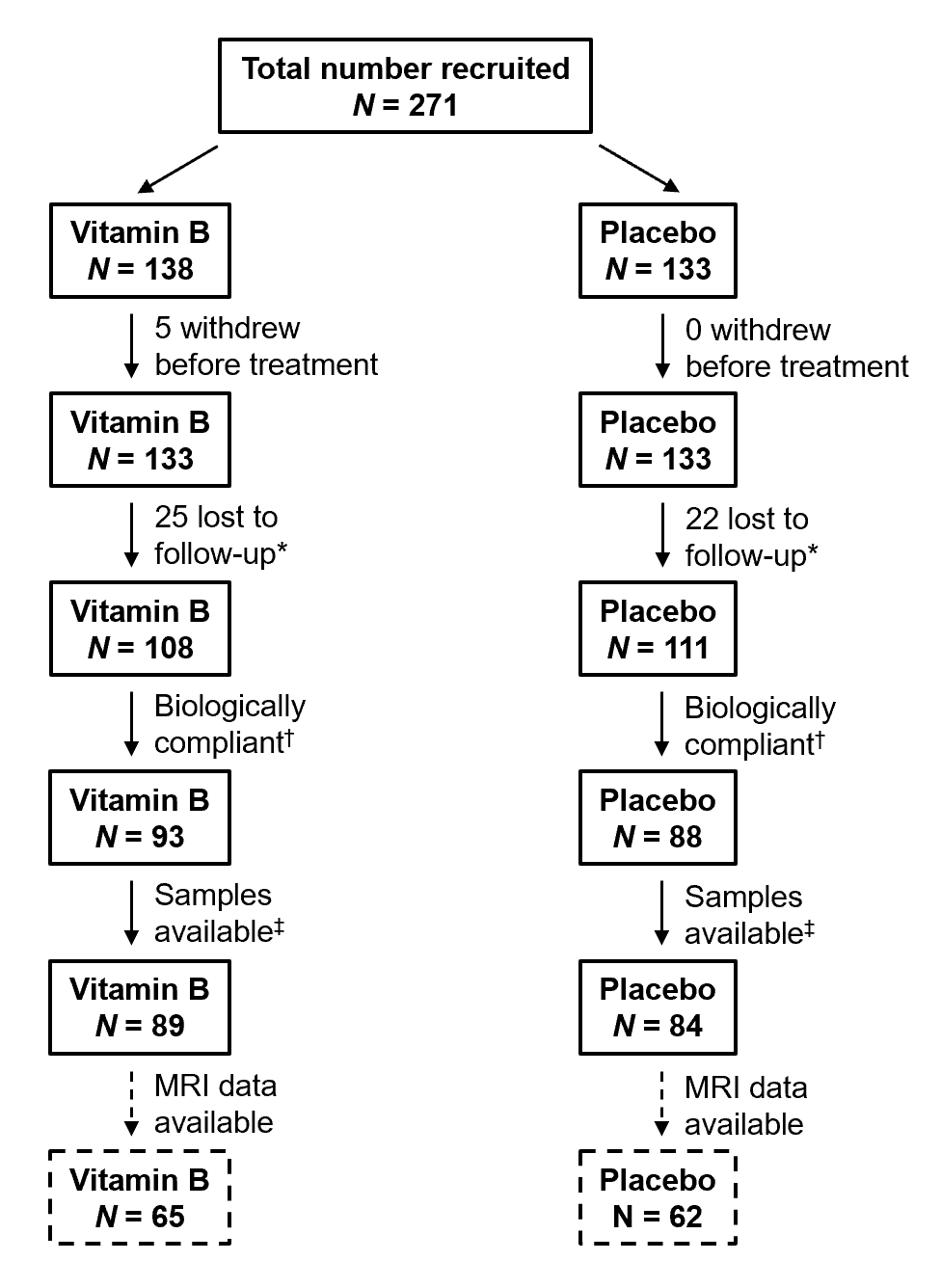
**

**SI Fig. 1** Participant flow through the trial (CONSORT diagram).^2^ "Available samples" refers to the samples remaining in the biobank for this retrospective study. In this study, all B vitamin and placebo samples (baseline and follow-up for each participant) were analysed. For specific analyses focused on brain atrophy, only participants with available MRI scans were included.

**Assessment of the potential confounding effect of treatment signature in the multivariate models**

B vitamins (B6, B12, and folate), aspirin and their selected derivatives were analysed across all LC-MS platforms. Due to their low serum concentrations, these compounds were not detectable by NMR. In most cases, the compounds were either not detected in the VITACOG samples or failed to meet the quality curation criteria. Where compounds did pass the curation criteria (e.g., salicylic acid), the corresponding signals were removed from the multivariate model to prevent bias.

**SI Table 1** List of parameters for B vitamins and homocysteine derivatives analysed using RPLC-MS (ESI^+^)

| Compound | Monoisotopic Molecular Weight | *Rt* (min) | *m/z* | Note |
| --- | --- | --- | --- | --- |
| Vitamin B6 | 169.0739 | 0.83, 1.17 | 170.0829 | / |
| Vitamin B12 | 1355.575 | 2.73, 3.21 | 678.2936 | [M+2H]^+^ |
| Folic acid | 441.1397 | 2.54, 3.17 | 442.1425 | / |
| Pyridoxal 5'-phosphate | 247.0246 | 0.86, 1.31 | 248.018 | / |
| Pyridoxal | 167.0582 | 0.84 | 150.5730, 168.0680 | [M-H_2_O-H]^+^ |
| 4-Pyridoxic acid | 183.0532 | 0.86, 1.67 | 166.0413, 184.0540 | [M-H_2_O-H]^+^ |
| Tetrahydrofolic acid | 445.171 | 0.86, 1.89 | 267.0818 | / |
| Homocysteine | 135.0354 | 0.79 | 136.04 | / |
| Cysteine | 121.0197 | 0.74 | 122.0306 | / |
| Salicylic acid | 138.0317 | 4.70 | 153.0562 | / |
| Acetylsalicylic acid | 180.0423 | 4.19 | 153.0562 | / |

**SI Table 2** List of parameters for B vitamins and homocysteine derivatives analysed using RPLC-MS (ESI^-^); some compounds were not detected (ND).

| Compound | Monoisotopic Molecular Weight | *Rt* (min) | *m/z* | Note |
| --- | --- | --- | --- | --- |
| Vitamin B6 | 169.0739 | 0.83, 1.17 | 168.8488 | / |
| Vitamin B12 | 1355.575 | ND | ND | / |
| Folic acid | 441.1397 | 2.54, 3.17 | 440.1307 | / |
| Pyridoxal 5'-phosphate | 247.0246 | 0.86, 1.31 | 246.0171 | / |
| Pyridoxal | 167.0582 | 0.84 | 166.0556 | / |
| 4-Pyridoxic acid | 183.0532 | 0.86, 1.67 | 182.0507 | / |
| Tetrahydrofolic acid | 445.171 | 0.86, 1.89 | 265.0859 | / |
| Homocysteine | 135.0354 | 0.79 | 134.0306 | / |
| Cysteine | 121.0197 | 0.74 | 120.0148 | / |
| Salicylic acid | 138.0317 | 4.70 | 137.0266 | / |
| Acetylsalicylic acid | 180.0423 | 4.19 | 137.0266 | / |

**SI Table 3** List of parameters for B vitamins and homocysteine derivatives analysed using AEC-MS (ESI^-^); some compounds were not detected (ND).

| Compound | Monoisotopic Molecular Weight | *Rt* (min) | *m/z* | Note |
| --- | --- | --- | --- | --- |
| Vitamin B6 | 169.0739 | 12.98 | 168.8472 | / |
| Vitamin B12 | 1355.575 | ND | ND | / |
| Folic acid | 441.1397 | ND | ND | / |
| Pyridoxal 5'-phosphate | 247.0246 | 21.5 | 246.0175 | / |
| Pyridoxal | 167.0582 | 17.27 | 166.0512 | / |
| 4-Pyridoxic acid | 183.0532 | 18.3 | 182.0462 | / |
| Tetrahydrofolic acid | 445.171 | ND | ND | / |
| Homocysteine | 135.0354 | ND | ND | / |
| Cysteine | 121.0197 | ND | ND | / |
| Salicylic acid | 138.0317 | 29.65 | 137.0247 | / |
| Acetylsalicylic acid | 180.0423 | ND | ND | / |

**Assessment of Potential Confounding Factors**

**Renal function**


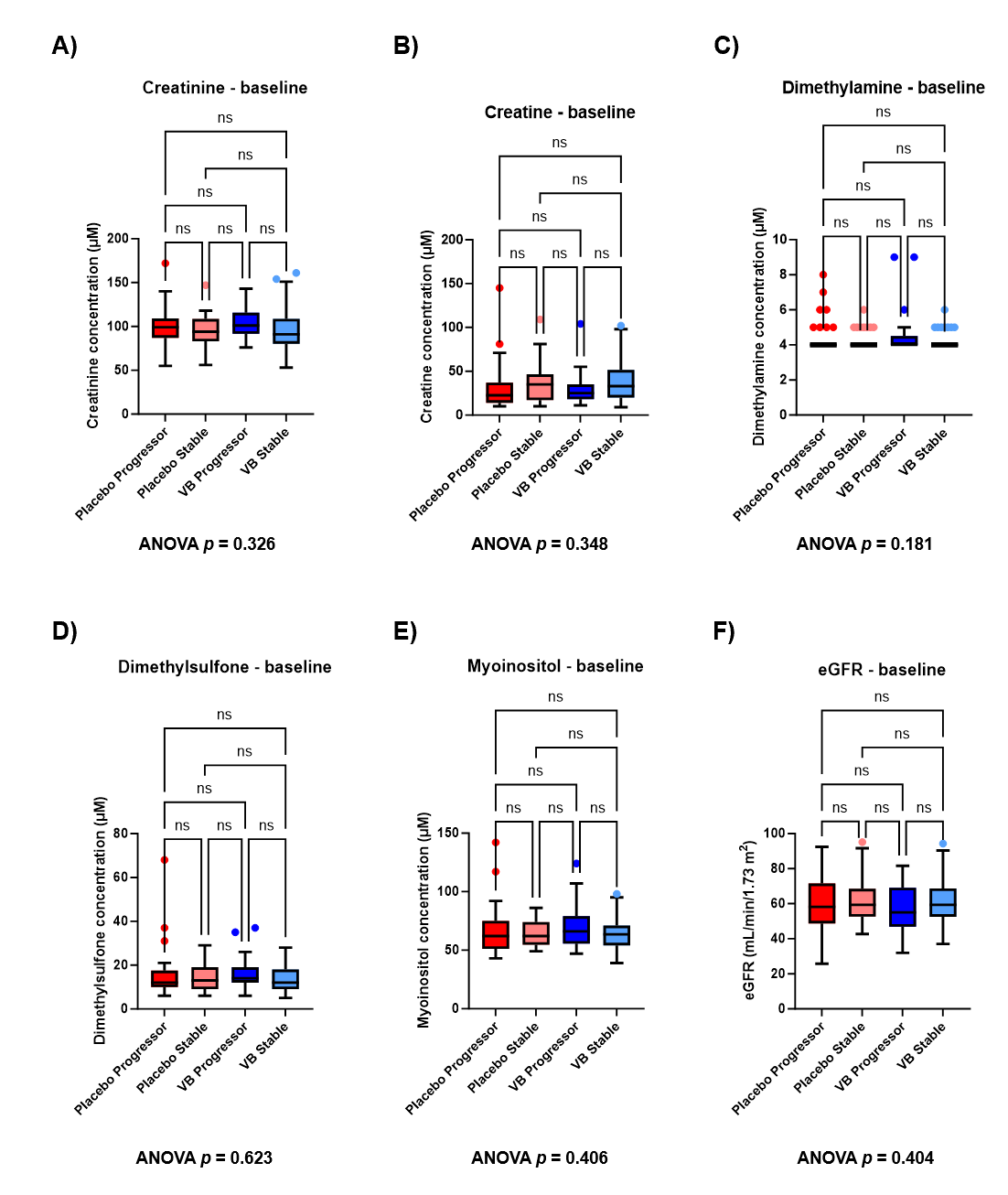


**SI Fig. 2 Assessment of renal and osmolyte markers at baseline across study groups.**
Boxplots show the distribution of serum **(A)** creatinine, **(B)** creatine, **(C)** dimethylamine, **(D)** dimethylsulfone, **(E)** myo-inositol, and **(F)** estimated glomerular filtration rate (eGFR) at baseline in placebo-progressors, placebo-stable, vitamin B (VB)-progressors, and VB-stable groups. No significant differences were observed between groups for any marker (ANOVA, all *p* > 0.18), indicating baseline comparability and supporting the absence of renal or osmotic confounding. eGFR was calculated using the CKD-EPI 2021 creatinine-based equation.

**Assessment of Potential Confounding Factors**


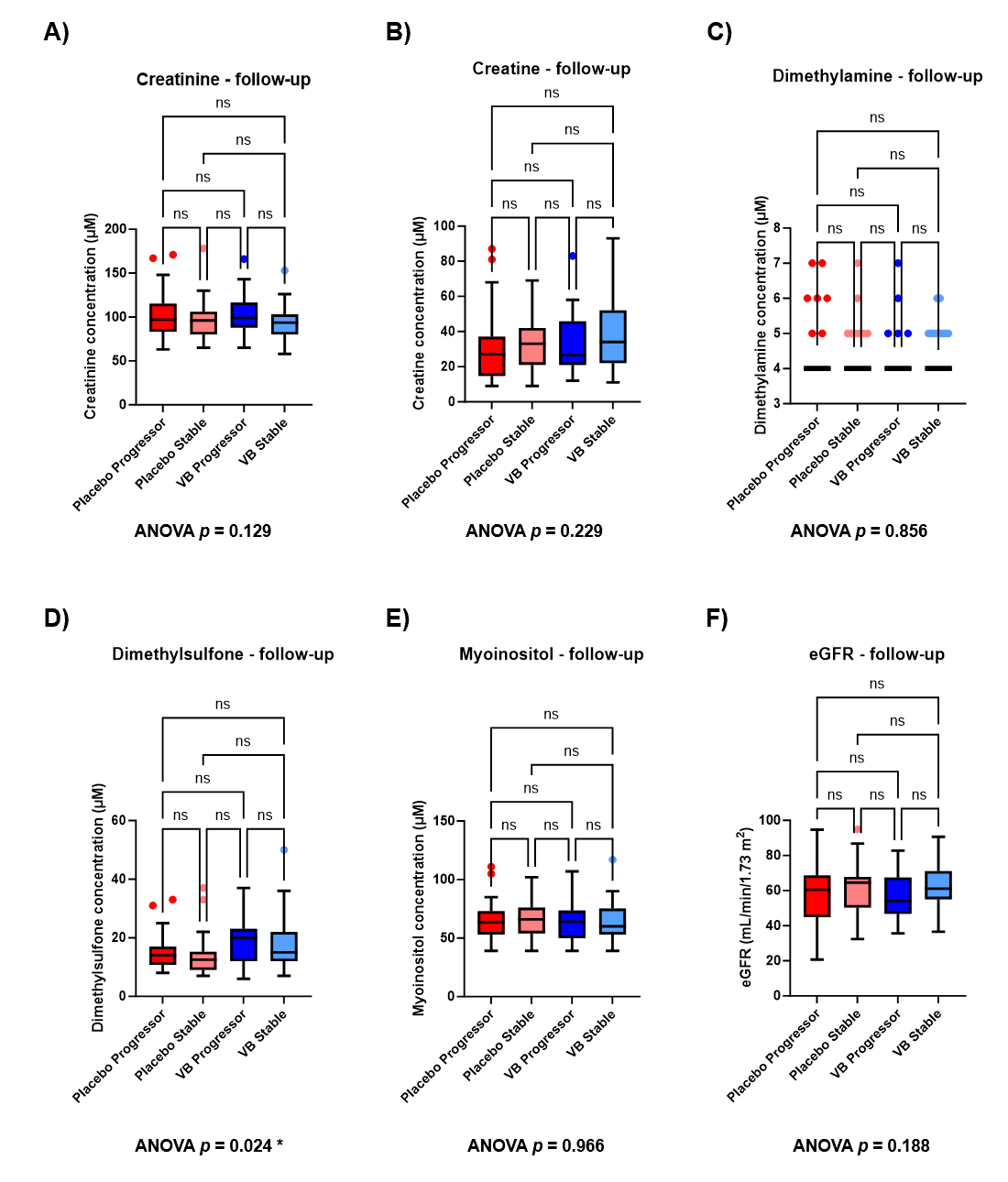


**SI Fig. 3 Assessment of renal and osmolyte markers at follow-up across study groups.** Boxplots show the distribution of serum **(A)** creatinine, **(B)** creatine, **(C)** dimethylamine, **(D)** dimethylsulfone, **(E)** myo-inositol, and **(F)** estimated glomerular filtration rate (eGFR) at follow-up in placebo-progressors, placebo-stable, vitamin B (VB)-progressors, and VB-stable groups. No significant differences were observed between groups for most markers (ANOVA, all *p* > 0.12), except for dimethylsulfone (*p* = 0.024), which showed a modest group effect. These findings support the absence of major renal or osmotic shifts following intervention. eGFR was calculated using the CKD-EPI 2021 creatinine-based equation.

**Assessment of Potential Confounding Factors**

***Central Nervous System (CNS) Targeting Drugs***


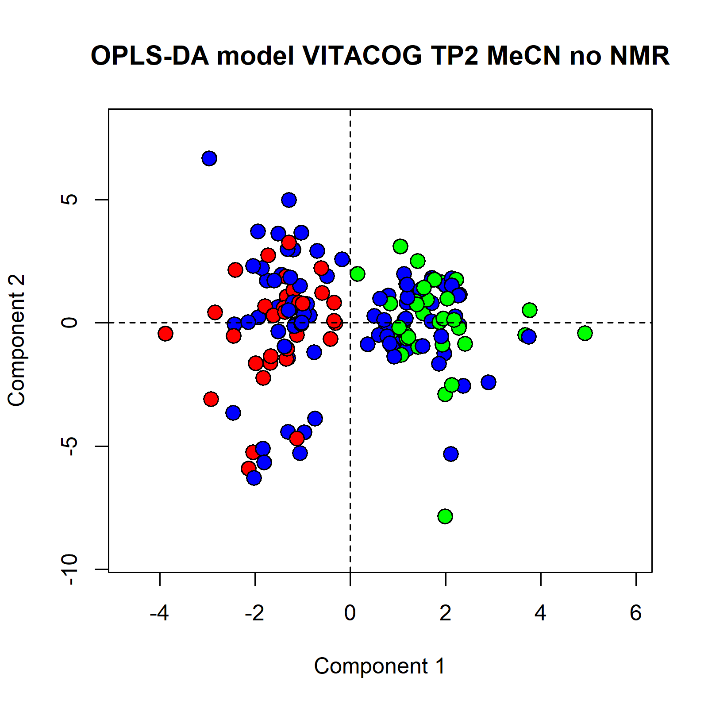


**SI Fig. 4.** OPLS-DA scores plot illustrating the distribution of individuals treated with B vitamins (green) and placebo controls (red), with those who were co-administered CNS targeting drugs highlighted in blue. The blue datapoints are evenly distributed across the plot, indicating no discernible clustering related to CNS targeting drugs administration.

***Cardiovascular System Targeting Drugs***

**
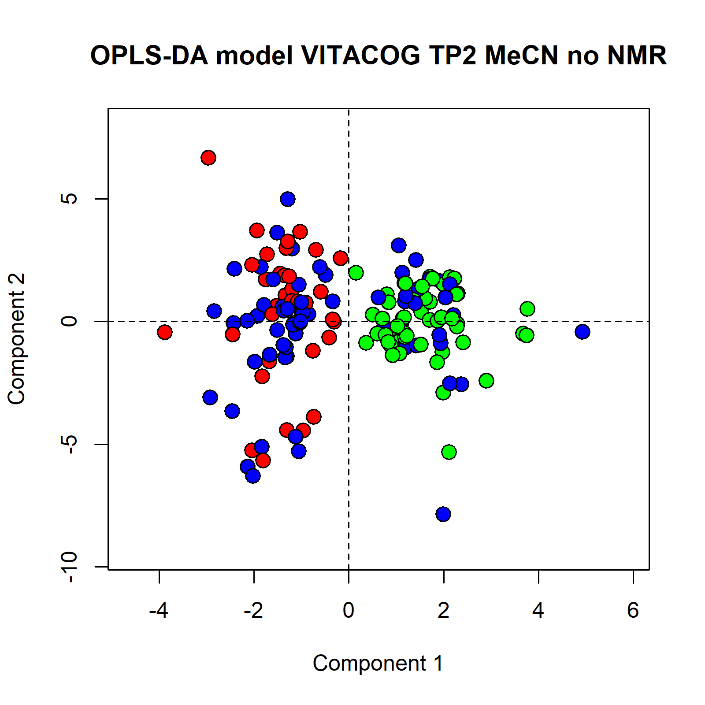
**

**SI Fig. 5** OPLS-DA scores plot illustrating the distribution of individuals treated with B vitamins (green) and placebo controls (red), with those who were co-administered cardiovascular system targeting drugs highlighted in blue. The blue datapoints are evenly distributed across the plot, indicating no discernible clustering related to cardiovascular system targeting drugs administration.

***Aspirin***


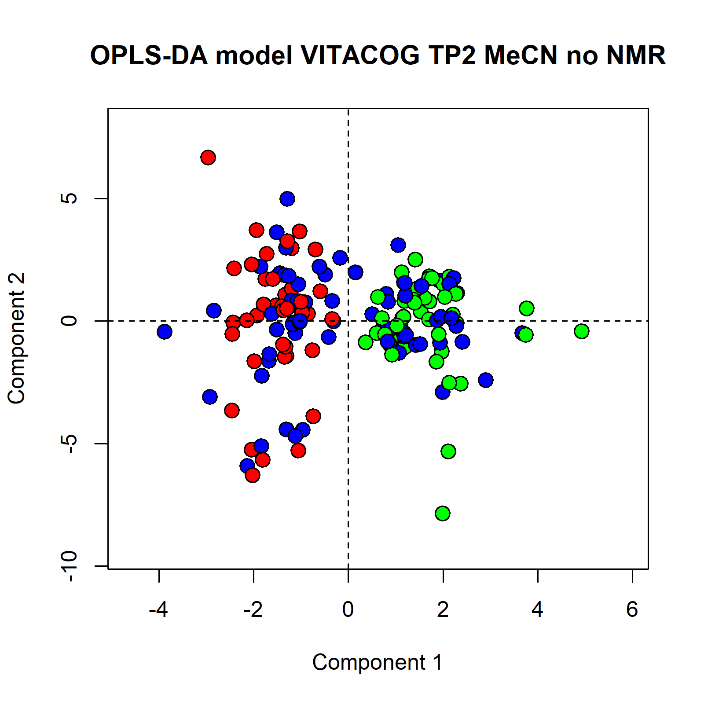


**SI Fig. 6** OPLS-DA scores plot illustrating the distribution of individuals treated with B vitamins (green) and placebo controls (red), with those who were co-administered aspirin highlighted in blue. The blue datapoints are evenly distributed across the plot, indicating no discernible clustering related to aspirin administration.

**Polar metabolome allows discriminating between B vitamin-treated individuals and placebo controls**


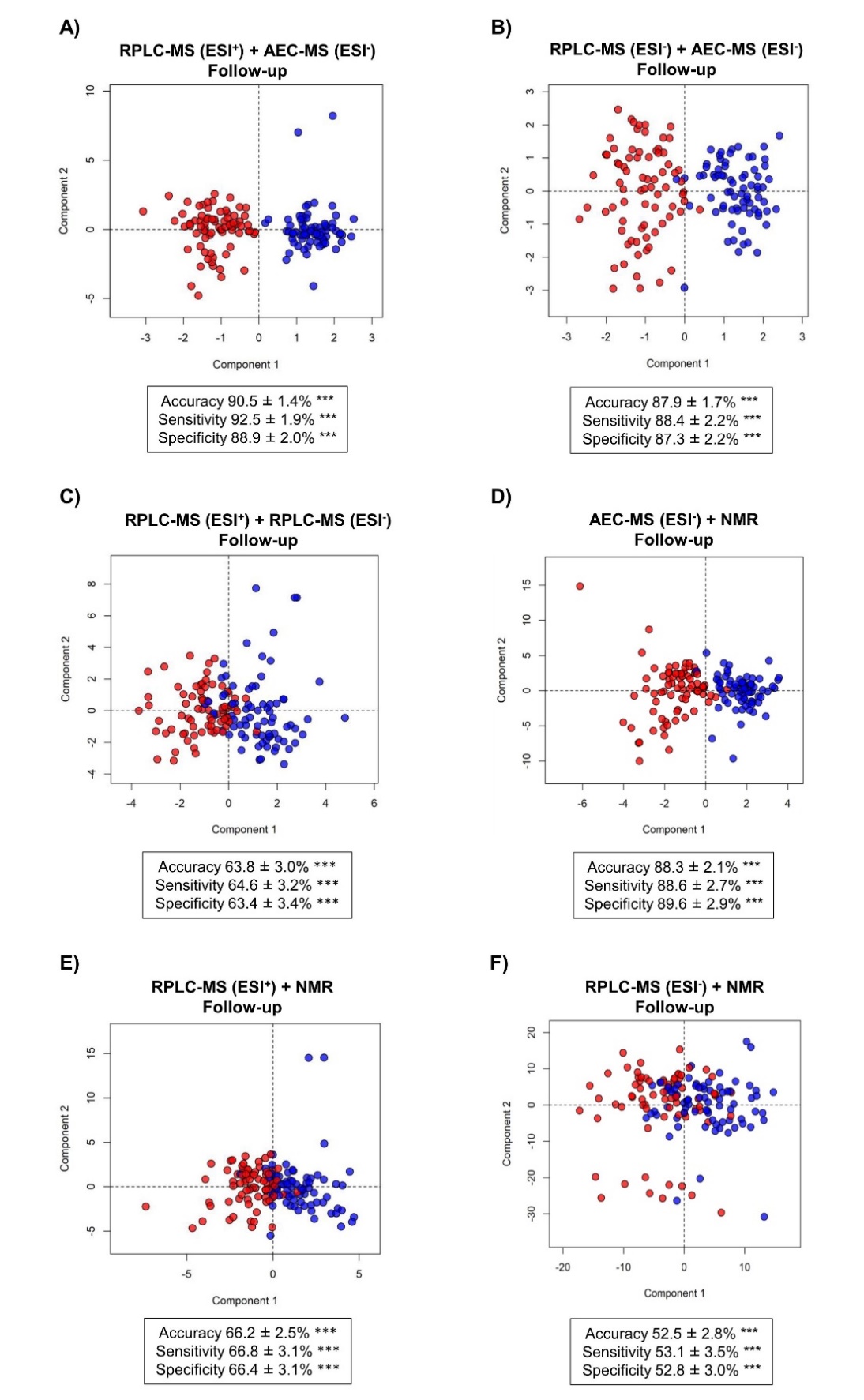


**SI Fig. 7** OPLS-DA score plots illustrating the separation between placebo-treated (red) and B vitamin-treated (blue) individuals based on serum metabolomic profiling at follow-up. Each panel displays results for a binary combination of analytical platforms: A) RPLC-MS (ESI⁺) + AEC-MS (ESI⁻); B) RPLC-MS (ESI⁺) + AEC-MS (ESI⁻) [duplicate of A to assess reproducibility]; C) RPLC-MS (ESI⁺) + RPLC-MS (ESI⁻); D) AEC-MS (ESI⁻) + NMR; E) RPLC-MS (ESI⁺) + NMR; F) RPLC-MS (ESI⁻) + NMR. Each model includes accuracy, sensitivity, and specificity (mean ± SD) derived from cross-validation. Significance (***p < 0.001) was assessed relative to a random class ensemble using the Kolmogorov–Smirnov test. These results highlight the dominant contribution of the AEC-MS platform and the incremental value of complementary platform integration.

**Polar metabolome allows discriminating between B vitamin-treated individuals and placebo controls**

**SI Table 4** List of compound-features and bins with the highest VIP score for the multiplatform metabolomics model discriminating between B vitamin-treated individuals and placebo controls at follow-up

| Feature | Frequency | Average Rank | Overall Score | Overall  Rank |
| --- | --- | --- | --- | --- |
| 9.49_372.2723 | 354 | 1.46 | 242.9 | 1 |
| 9.68_261.1482 | 858 | 3.66 | 234.2 | 2 |
| 9.68_239.1683 | 989 | 5.02 | 197.0 | 3 |
| 8.52_557.2365 | 919 | 4.93 | 186.5 | 4 |
| Mannitol | 298 | 1.92 | 155.5 | 5 |
| 7.39_229.1446 | 995 | 7.76 | 128.2 | 6 |
| 10.18_726.3464 | 642 | 5.93 | 108.3 | 7 |
| 0.77_365.1172 | 983 | 11.43 | 86.0 | 8 |
| 4.39_198.0581 | 1000 | 13.34 | 74.9 | 9 |
| 8.26_539.2501 | 314 | 4.65 | 67.6 | 10 |
| 5.54_360.2393 | 909 | 13.70 | 66.3 | 11 |
| 4.39_260.0292 | 964 | 15.82 | 60.9 | 12 |
| 10.01_491.8127 | 457 | 7.97 | 57.3 | 13 |
| 12.73_209.0820 | 605 | 10.79 | 56.1 | 14 |
| [2.95....2.97] | 423 | 7.67 | 55.1 | 15 |
| 4.77_266.9830 | 160 | 2.98 | 53.7 | 16 |
| 7.96_449.3099 | 583 | 12.25 | 47.6 | 17 |
| Succinic acid | 895 | 20.13 | 44.5 | 18 |
| 5.75_479.2639 | 524 | 11.97 | 43.8 | 19 |
| 5.49_427.1624 | 123 | 3.06 | 40.2 | 20 |
| 8.80_453.2498 | 819 | 20.36 | 40.2 | 21 |
| 13.84_452.0498 | 834 | 21.89 | 38.1 | 22 |
| 4.58_857.2492 | 890 | 24.00 | 37.1 | 23 |
| 3.62_121.0325 | 533 | 16.31 | 32.7 | 24 |
| 4.58_857.0491 | 949 | 29.11 | 32.6 | 25 |
|  |  |  |  |  |

**Polar metabolome allows discriminating between B vitamin-treated individuals and placebo controls**

**SI Table 5** List of identified LC-MS-detected metabolites with the highest VIP scores for the multiplatform metabolomics model discriminating between B vitamin-treated individuals and placebo controls at follow-up. Note that only identified metabolites were used construct the used multivariate model constructed model accuracy: 63.5 ± 2.8%).

†† RPLC-MS (pos. ion mode); † RPLC-MS (neg. ion mode); no symbol AEC-MS (neg. ion mode).

| Metabolite | Frequency | Average  Rank | Overall  Score | Overall  Rank |
| --- | --- | --- | --- | --- |
| Succinic acid | 785 | 2.86 | 274.86 | 1 |
| Arabitol | 877 | 3.44 | 255.27 | 2 |
| Lactose | 970 | 4.44 | 218.51 | 3 |
| Sugar-phosphate | 997 | 7.06 | 141.25 | 4 |
| Mannitol | 808 | 7.82 | 103.33 | 5 |
| α-Ketoglutaric acid | 994 | 14.47 | 68.69 | 6 |
| 3,4-Dihydroxybenzylamine | 982 | 17.41 | 56.40 | 7 |
| α-Ketobutyric acid | 159 | 2.96 | 53.79 | 8 |
| 2-Hydroxyhexanoic acid | 993 | 19.19 | 51.73 | 9 |
| Glucose | 955 | 18.60 | 51.35 | 10 |
| Ribulose | 983 | 19.89 | 49.42 | 11 |
| 2-Hydroxybutyric acid | 961 | 19.78 | 48.58 | 12 |
| ††Propionyl carnitine | 244 | 5.03 | 48.48 | 13 |
| Glycolic acid | 323 | 7.11 | 45.46 | 14 |
| Quinolinic acid | 913 | 20.80 | 43.90 | 15 |
| 2-Methylcitric acid | 513 | 11.75 | 43.66 | 16 |
| Aspartate | 965 | 22.19 | 43.48 | 17 |
| †Citraconic acid | 506 | 13.85 | 36.52 | 18 |
| Pyruvic acid | 759 | 21.05 | 36.06 | 19 |
| 3,3-Dimethylglutaric acid | 827 | 23.79 | 34.76 | 20 |
| Acetoacetate | 850 | 26.15 | 32.51 | 21 |
| Linoleic acid | 696 | 21.42 | 32.49 | 22 |
| *N*-acetylglutamic acid | 910 | 28.68 | 31.73 | 23 |
| Sorbitol | 894 | 31.37 | 28.50 | 24 |
| 4-Methyl-2-oxovaleric acid | 690 | 29.05 | 23.75 | 25 |

**Three-way ANOVA reveals B vitamin-dependent changes and their association with brain atrophy**


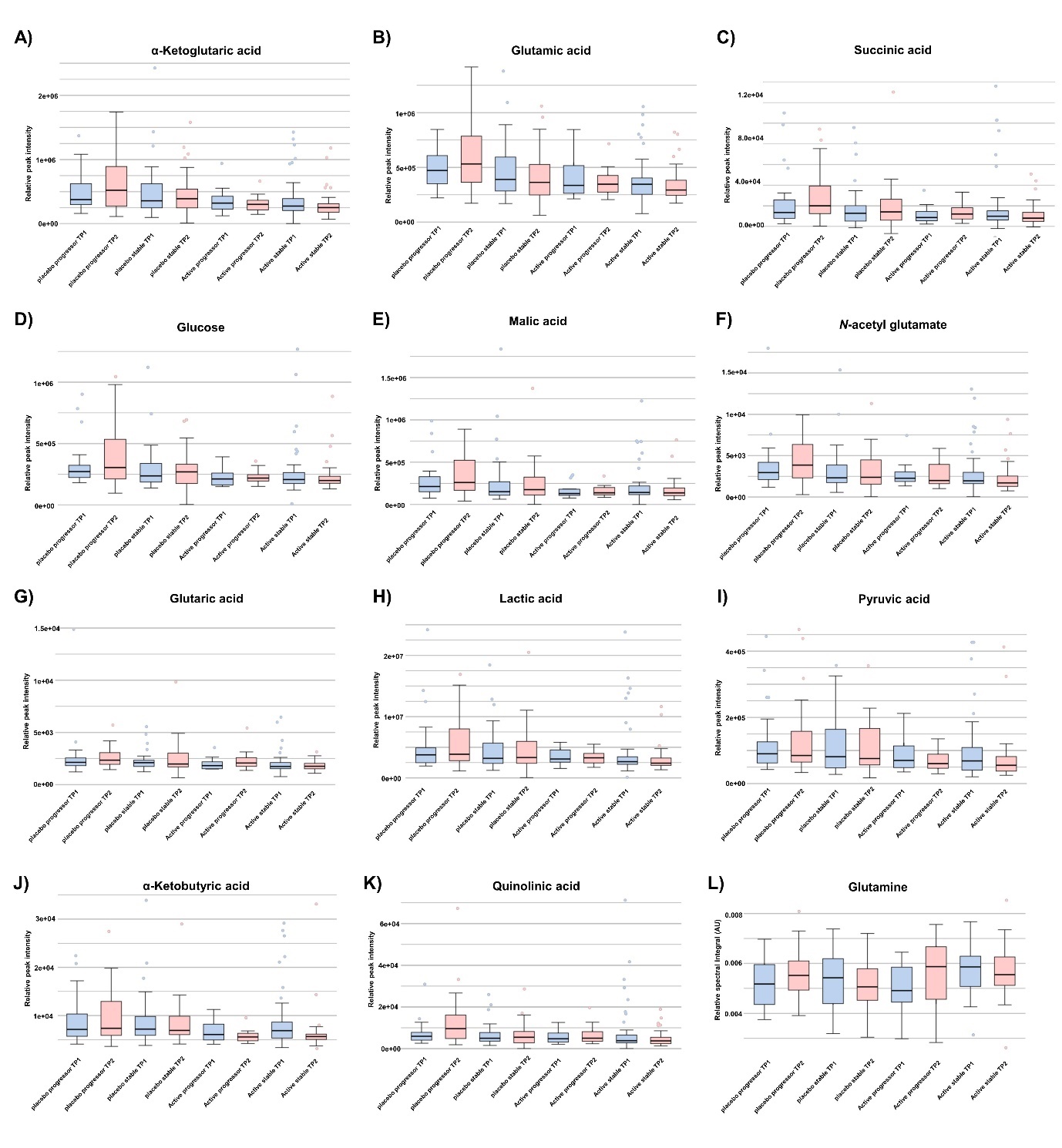


**SI Fig. 8** Three-way ANOVA assessing the effects of B vitamin treatment (active vs placebo), atrophy progression (stable vs progressor), and temporal effects (TP1 (baseline) vs TP2 (follow-up) in the VITACOG cohort. **A) 2-oxoglutaric acid:** treatment effect *p* < 0.0001 (0.002); atrophy effect *p* = 0.10 (0.46) ; timepoint effect *p* = 0.23 (0.62); **B) glutamic acid:** treatment effect *p* < 0.0001 (0.0004); atrophy effect *p* = 0.03 (0.21); timepoint effect *p* = 0.59 (0.85); **C) succinic acid:** treatment effect *p* < 0.0015 (0.03); atrophy effect *p* = 0.18 (0.55); timepoint effect *p* = 0.41 (0.75); **D) glucose:** treatment effect *p* = 0.0001 (0.008); atrophy effect *p* = 0.07 (0.39); timepoint effect *p* = 0.40 (0.75); **E) malic acid:** treatment effect *p* = 0.0001 (0.008); atrophy effect *p* = 0.21 (0.59); timepoint effect *p* = 0.28 (0.65); **F) *N*-acetylglutamate:** treatment effect *p* = 0.0004 (0.01); atrophy effect *p* = 0.03 (0.25); timepoint effect *p* = 0.27 (0.64); **G) glutaric acid:** treatment effect *p* = 0.0002 (0.009); atrophy effect *p* = 0.10 (0.46); timepoint effect *p* = 0.69 (0.91); **H) lactic acid:** treatment effect *p* = 0.0004 (0.01); atrophy effect *p* = 0.12 (0.50); timepoint effect *p* = 0.16 (0.54); **I) pyruvic acid:** treatment effect *p* = 0.001 (0.02); atrophy effect *p* = 0.14 (0.52); timepoint effect *p* = 0.10 (0.44); **J) 2-ketobutyric acid:** treatment effect *p* = 0.002 (0.03) ; atrophy effect *p* = 0.62 (0.87); timepoint effect *p* = 0.03 (0.23); **K) quinolinic acid:** treatment effect *p* =0.02 (0.19); atrophy effect *p* = 0.03 (0.24); timepoint effect *p* = 0.27 (.065); **L) glutamine:** treatment effect *p* = 0.04; atrophy effect *p* = 0.67; timepoint effect *p* = 0.57. One outlier was excluded from the graphical representation to enhance data clarity. This exclusion did not significantly impact the results.

**Three-way ANOVA reveals B vitamin-dependent changes and their association with brain atrophy**

**SI Table 6** List of LC-MS identified metabolites and results of three-way ANOVA (main class effects).

†† RPLC-MS (pos. ion mode); † RPLC-MS (neg. ion mode); no symbol AEC-MS (neg. ion mode).

| Metabolite | Class 1 | | Class 2 | | Class 3 | |
| --- | --- | --- | --- | --- | --- | --- |
|  | *p*-value | FDR | *p*-value | FDR | *p*-value | FDR |
| †2-*C*-Methyl-*D*-Erythritol-4-phosphate | 0.3349 | 0.6897 | 0.6486 | 0.8965 | 0.7487 | 0.9248 |
| 2-Hydroxybutyric acid | 0.0061 | 0.0641 | 0.6330 | 0.8862 | 0.0477 | 0.3186 |
| 2-Hydroxyhexanoic acid | 0.0823 | 0.4290 | 0.8176 | 0.9466 | 0.0178 | 0.1596 |
| 2-Ketobutyric acid | 0.0017 | 0.0275 | 0.6178 | 0.8749 | 0.0295 | 0.2299 |
| 2-Ketoglutaric acid | <0.0001 | 0.0021 | 0.0996 | 0.4594 | 0.2346 | 0.6159 |
| 2-Methylcitric acid | 0.0035 | 0.0422 | 0.1484 | 0.5267 | 0.1180 | 0.5026 |
| 3,3-Dimethyl glutarate | 0.0032 | 0.0395 | 0.2172 | 0.5962 | 0.1261 | 0.5034 |
| 3,4-Dihydroxybenzylamine | 0.0026 | 0.0341 | 0.2532 | 0.6347 | 0.4190 | 0.7529 |
| 3,4-Dihydroxyphenylacetic acid | 0.2342 | 0.6159 | 0.3583 | 0.7014 | 0.6554 | 0.8965 |
| 3-Dehydroquinate | 0.0021 | 0.0302 | 0.2608 | 0.6371 | 0.2909 | 0.6526 |
| 3-Ethylmalate | 0.0009 | 0.0185 | 0.0712 | 0.3969 | 0.2286 | 0.6134 |
| 3-Hydroxyisovaleric acid | 0.0003 | 0.0090 | 0.1311 | 0.5082 | 0.3802 | 0.7261 |
| 3-Isopropylmalic acid | 0.0043 | 0.0499 | 0.0833 | 0.4290 | 0.2249 | 0.6060 |
| 3-Methoxyphenylacetic acid | 0.0046 | 0.0513 | 0.0155 | 0.1404 | 0.4651 | 0.7808 |
| 4-Methoxybenzoic acid | 0.0046 | 0.0513 | 0.5788 | 0.8468 | 0.9646 | 0.9940 |
| 5-Methoxyindoleacetate | 0.1466 | 0.5267 | 0.1034 | 0.4699 | 0.4480 | 0.7707 |
| Acetoacetate | 0.0268 | 0.2149 | 0.2071 | 0.5838 | 0.4157 | 0.7529 |
| Acetylglycine | 0.0951 | 0.4488 | 0.2809 | 0.6526 | 0.2405 | 0.6179 |
| Adonitol | 0.0003 | 0.0090 | 0.1938 | 0.5757 | 0.5231 | 0.8224 |
| Allantoin | 0.0003 | 0.0090 | 0.0404 | 0.2830 | 0.3426 | 0.6924 |
| α-Ketoisovaleric acid | <0.0001 | 0.0021 | 0.3924 | 0.7358 | 0.1053 | 0.4752 |
| Arabinonic acid | 0.0018 | 0.0281 | 0.1564 | 0.5380 | 0.1596 | 0.5401 |
| Arabitol | <0.0001 | 0.0034 | 0.0138 | 0.1292 | 0.6639 | 0.8994 |
| Ascorbate | 0.0026 | 0.0341 | 0.1543 | 0.5374 | 0.3301 | 0.6897 |
| ††Aspartame | 0.0243 | 0.2031 | 0.1203 | 0.5026 | 0.5995 | 0.8591 |
| Aspartate | 0.0010 | 0.0185 | 0.2658 | 0.6409 | 0.1820 | 0.5626 |
| ††Butyrylcarnitine | 0.1706 | 0.5474 | 0.4550 | 0.7762 | 0.8548 | 0.9613 |
| †Citraconic acid | 0.7684 | 0.9279 | 0.9436 | 0.9895 | 0.5209 | 0.8224 |
| Citramalic acid | 0.0002 | 0.0083 | 0.1890 | 0.5641 | 0.4286 | 0.7573 |
| Citric acid | 0.0129 | 0.1228 | 0.3738 | 0.7226 | 0.2020 | 0.5838 |
| †Deoxycholic acid | 0.9806 | 0.9953 | 0.8044 | 0.9367 | 0.7333 | 0.9189 |
| ††Dopamine hydrochloride | 0.9074 | 0.9780 | 0.1871 | 0.5641 | 0.3232 | 0.6875 |
| Ethylmalonic acid | 0.0369 | 0.2645 | 0.3445 | 0.6924 | 0.1661 | 0.5468 |
| Fructose | 0.0010 | 0.0185 | 0.2897 | 0.6526 | 0.5812 | 0.8468 |
| Galactaric acid | 0.0007 | 0.0157 | 0.2813 | 0.6526 | 0.4518 | 0.7750 |
| Galacturonic acid | 0.0442 | 0.3056 | 0.4398 | 0.7609 | 0.2396 | 0.6179 |
| Gluconate | 0.0024 | 0.0322 | 0.1373 | 0.5195 | 0.2054 | 0.5838 |
| Gluconolactone | 0.0067 | 0.0689 | 0.1496 | 0.5267 | 0.3513 | 0.6991 |
| Glucose | 0.0001 | 0.0079 | 0.0654 | 0.3903 | 0.4014 | 0.7466 |
| Glucose-1-phosphate | 0.0013 | 0.0227 | 0.3242 | 0.6875 | 0.0764 | 0.4079 |
| Glucuronic acid | 0.0019 | 0.0281 | 0.2173 | 0.5962 | 0.1688 | 0.5474 |
| Glutamic acid | <0.0001 | 0.0004 | 0.0261 | 0.2116 | 0.5879 | 0.8505 |
| Glutaric acid | 0.0002 | 0.0087 | 0.0987 | 0.4589 | 0.6934 | 0.9091 |
| Glyceric acid | 0.0031 | 0.0393 | 0.3381 | 0.6907 | 0.3058 | 0.6722 |
| Glycolic acid | 0.0003 | 0.0094 | 0.2327 | 0.6159 | 0.3394 | 0.6907 |
| Isocitrate | 0.0195 | 0.1724 | 0.2046 | 0.5838 | 0.0891 | 0.4371 |
| Lactic acid | 0.0004 | 0.0104 | 0.1217 | 0.5026 | 0.1622 | 0.5427 |
| Lactose | 0.1863 | 0.5641 | 0.6548 | 0.8965 | 0.7925 | 0.9354 |
| †Linoleic Acid | 0.7065 | 0.9091 | 0.1958 | 0.5788 | 0.2095 | 0.5853 |
| †*L*-Theanine | 0.7648 | 0.9279 | 0.8779 | 0.9661 | 0.0115 | 0.1116 |
| Malic acid | 0.0001 | 0.0079 | 0.2089 | 0.5853 | 0.2825 | 0.6526 |
| Malitol | 0.0014 | 0.0236 | 0.0059 | 0.0632 | 0.8909 | 0.9741 |
| Mannitol | 0.0001 | 0.0045 | 0.0949 | 0.4488 | 0.4075 | 0.7466 |
| Mannose | 0.0004 | 0.0101 | 0.2960 | 0.6570 | 0.5431 | 0.8246 |
| Methylglutaric acid | 0.0152 | 0.1400 | 0.0690 | 0.3969 | 0.2416 | 0.6179 |
| Methyl-phenylacetate | 0.1175 | 0.5026 | 0.7895 | 0.9354 | 0.9389 | 0.9895 |
| Mytilitol | 0.0004 | 0.0104 | 0.1444 | 0.5267 | 0.2024 | 0.5838 |
| *N*-Acetylaspartate | 0.0004 | 0.0104 | 0.0385 | 0.2724 | 0.5087 | 0.8175 |
| *N*-Acetylglutamate | 0.0004 | 0.0104 | 0.0343 | 0.2549 | 0.2681 | 0.6409 |
| *N*-Acetyl-L-alanine | 0.0010 | 0.0185 | 0.1297 | 0.5082 | 0.3434 | 0.6924 |
| ††*N*-Acetyl-*L*-methionine | 0.1596 | 0.5401 | 0.1736 | 0.5534 | 0.7783 | 0.9330 |
| *N*-Acetylneuraminic acid | 0.0069 | 0.0698 | 0.2055 | 0.5838 | 0.1242 | 0.5026 |
| Nonate | 0.0101 | 0.0996 | 0.2684 | 0.6409 | 0.3231 | 0.6875 |
| Oxalic acid | 0.0023 | 0.0322 | 0.3582 | 0.7014 | 0.4222 | 0.7529 |
| Pantoic acid | 0.0496 | 0.3211 | 0.5785 | 0.8468 | 0.8071 | 0.9381 |
| Pantothenic acid | 0.0019 | 0.0281 | 0.3403 | 0.6907 | 0.4358 | 0.7609 |
| *p*-Hydroxyphenylacetic acid | 0.9422 | 0.9895 | 0.9508 | 0.9895 | 0.1705 | 0.5474 |
| Phytanic acid | 0.9943 | 0.9976 | 0.8597 | 0.9613 | 0.4672 | 0.7817 |
| ††Propionylcarnitine | 0.8977 | 0.9744 | 0.6817 | 0.9091 | 0.7954 | 0.9364 |
| Protocatechuic acid | 0.0002 | 0.0083 | 0.5411 | 0.8246 | 0.9145 | 0.9804 |
| Pyroglutamic acid | 0.0059 | 0.0632 | 0.2199 | 0.5978 | 0.1159 | 0.5026 |
| Pyruvic acid | 0.0010 | 0.0185 | 0.1391 | 0.5229 | 0.0974 | 0.4562 |
| Quinic acid | 0.0040 | 0.0475 | 0.3251 | 0.6875 | 0.7591 | 0.9279 |
| Quinolinic acid | 0.0235 | 0.1991 | 0.0369 | 0.2645 | 0.2865 | 0.6526 |
| Raffinose | 0.1102 | 0.4937 | 0.4881 | 0.8012 | 0.2139 | 0.5921 |
| Ribulose | 0.0002 | 0.0087 | 0.0254 | 0.2088 | 0.3074 | 0.6735 |
| Sorbitol | 0.0021 | 0.0302 | 0.1809 | 0.5620 | 0.4864 | 0.8005 |
| †Stearic acid | 0.7423 | 0.9208 | 0.1708 | 0.5474 | 0.2761 | 0.6493 |
| Succinic acid | 0.0015 | 0.0255 | 0.1749 | 0.5546 | 0.4053 | 0.7466 |
| Tartaric acid | 0.0481 | 0.3186 | 0.1452 | 0.5267 | 0.1782 | 0.5620 |
| Taurine | 0.0018 | 0.0281 | 0.1318 | 0.5082 | 0.0855 | 0.4304 |
| ††Theophylline | 0.6534 | 0.8965 | 0.9687 | 0.9948 | 0.8489 | 0.9613 |
| †Tryptamine | 0.7161 | 0.9112 | 0.9538 | 0.9895 | 0.8532 | 0.9613 |
| Uridine | 0.0002 | 0.0083 | 0.0574 | 0.3569 | 0.4077 | 0.7466 |
| ††Vanillin | 0.1314 | 0.5082 | 0.7921 | 0.9354 | 0.7223 | 0.9112 |
| Xylulose | 0.0009 | 0.0185 | 0.0845 | 0.4290 | 0.3243 | 0.6875 |

**Three-way ANOVA reveals B vitamin-dependent changes and their association with brain atrophy**

**SI Table 7** List of LC-MS identified metabolites and results of three-way ANOVA (classes interactions).

†† RPLC-MS (pos. ion mode); † RPLC-MS (neg. ion mode); no symbol AEC-MS (neg. ion mode).

| Metabolite | Class 1:2 interaction | | Class 1:3 interaction | | Class 2:3 interaction | |
| --- | --- | --- | --- | --- | --- | --- |
|  | *p*-value | FDR | *p*-value | FDR | *p*-value | FDR |
| †2-*C*-Methyl-*D*-Erythritol-4-phosphate | 0.5424 | 0.8246 | 0.5350 | 0.8246 | 0.5387 | 0.8246 |
| 2-Hydroxybutyric acid | 0.1461 | 0.5267 | 0.3468 | 0.6940 | 0.2902 | 0.6526 |
| 2-Hydroxyhexanoic acid | 0.0494 | 0.3211 | 0.9008 | 0.9744 | 0.0348 | 0.2554 |
| 2-Ketobutyric acid | 0.0723 | 0.3969 | 0.4933 | 0.8055 | 0.7421 | 0.9208 |
| 2-Ketoglutaric acid | 0.0941 | 0.4488 | 0.5026 | 0.8138 | 0.9183 | 0.9811 |
| 2-Methylcitric acid | 0.1982 | 0.5804 | 0.8499 | 0.9613 | 0.9507 | 0.9895 |
| 3,3-Dimethyl glutarate | 0.1646 | 0.5447 | 0.9526 | 0.9895 | 0.7697 | 0.9279 |
| 3,4-Dihydroxybenzylamine | 0.2586 | 0.6371 | 0.5333 | 0.8246 | 0.4650 | 0.7808 |
| 3,4-Dihydroxyphenylacetic acid | 0.0649 | 0.3903 | 0.8800 | 0.9661 | 0.8513 | 0.9613 |
| 3-Dehydroquinate | 0.0879 | 0.4351 | 0.8964 | 0.9744 | 0.8580 | 0.9613 |
| 3-Ethylmalate | 0.2543 | 0.6347 | 0.9871 | 0.9957 | 0.7008 | 0.9091 |
| 3-Hydroxyisovaleric acid | 0.0734 | 0.3991 | 0.2851 | 0.6526 | 0.2721 | 0.6448 |
| 3-Isopropylmalic acid | 0.1486 | 0.5267 | 0.9875 | 0.9957 | 0.7900 | 0.9354 |
| 3-Methoxyphenylacetic acid | 0.4208 | 0.7529 | 0.6615 | 0.8992 | 0.5281 | 0.8246 |
| 4-Methoxybenzoic acid | 0.7635 | 0.9279 | 0.9003 | 0.9744 | 0.4829 | 0.7988 |
| 5-Methoxyindoleacetate | 0.6891 | 0.9091 | 0.1793 | 0.5620 | 0.3214 | 0.6875 |
| Acetoacetate | 0.0568 | 0.3567 | 0.0472 | 0.3186 | 0.1236 | 0.5026 |
| Acetylglycine | 0.0602 | 0.3702 | 0.8039 | 0.9367 | 0.5443 | 0.8246 |
| Adonitol | 0.0510 | 0.3236 | 0.9941 | 0.9976 | 0.2967 | 0.6570 |
| Allantoin | 0.0306 | 0.2360 | 0.9601 | 0.9917 | 0.6574 | 0.8965 |
| α-Ketoisovaleric acid | 0.1360 | 0.5195 | 0.2567 | 0.6356 | 0.5429 | 0.8246 |
| Arabinonic acid | 0.1496 | 0.5267 | 0.7376 | 0.9206 | 0.9000 | 0.9744 |
| Arabitol | 0.9209 | 0.9822 | 0.3323 | 0.6897 | 0.6397 | 0.8915 |
| Ascorbate | 0.5010 | 0.8138 | 0.7798 | 0.9330 | 0.8820 | 0.9661 |
| ††Aspartame | 0.4356 | 0.7654 | 0.8317 | 0.9538 | 0.4072 | 0.7466 |
| Aspartate | 0.1213 | 0.5026 | 0.4325 | 0.7591 | 0.5761 | 0.8468 |
| ††Butyrylcarnitine | 0.0002 | 0.0083 | 0.6228 | 0.8779 | 0.8653 | 0.9634 |
| †Citraconic acid | 0.2673 | 0.6409 | 0.2312 | 0.6148 | 0.2561 | 0.6356 |
| Citramalic acid | 0.0897 | 0.4371 | 0.5218 | 0.8224 | 0.3787 | 0.7261 |
| Citric acid | 0.8722 | 0.9657 | 0.3032 | 0.6690 | 0.3317 | 0.6897 |
| †Deoxycholic acid | 0.1367 | 0.5195 | 0.4396 | 0.7609 | 0.0909 | 0.4395 |
| ††Dopamine hydrochloride | 0.0718 | 0.3969 | 0.7052 | 0.9091 | 0.5651 | 0.8393 |
| Ethylmalonic acid | 0.1672 | 0.5474 | 0.6268 | 0.8797 | 0.5068 | 0.8165 |
| Fructose | 0.0321 | 0.2447 | 0.7473 | 0.9248 | 0.2602 | 0.6371 |
| Galactaric acid | 0.0508 | 0.3236 | 0.1882 | 0.5641 | 0.5928 | 0.8534 |
| Galacturonic acid | 0.4150 | 0.7529 | 0.8777 | 0.9661 | 0.9437 | 0.9895 |
| Gluconate | 0.1300 | 0.5082 | 0.8101 | 0.9398 | 0.7314 | 0.9183 |
| Gluconolactone | 0.1544 | 0.5374 | 0.7606 | 0.9279 | 0.7085 | 0.9091 |
| Glucose | 0.0283 | 0.2239 | 0.9042 | 0.9764 | 0.7078 | 0.9091 |
| Glucose-1-phosphate | 0.2425 | 0.6179 | 0.5016 | 0.8138 | 0.5597 | 0.8375 |
| Glucuronic acid | 0.3352 | 0.6897 | 0.9160 | 0.9804 | 0.7250 | 0.9122 |
| Glutamic acid | 0.3117 | 0.6804 | 0.2515 | 0.6347 | 0.2368 | 0.6179 |
| Glutaric acid | 0.7091 | 0.9091 | 0.9770 | 0.9953 | 0.6202 | 0.8763 |
| Glyceric acid | 0.2296 | 0.6134 | 0.2914 | 0.6526 | 0.7172 | 0.9112 |
| Glycolic acid | 0.2188 | 0.5976 | 0.5201 | 0.8224 | 0.5708 | 0.8417 |
| Isocitrate | 0.1246 | 0.5026 | 0.9229 | 0.9826 | 0.7860 | 0.9354 |
| Lactic acid | 0.9943 | 0.9976 | 0.8943 | 0.9744 | 0.8566 | 0.9613 |
| Lactose | 0.1265 | 0.5034 | 0.8392 | 0.9606 | 0.8009 | 0.9367 |
| †Linoleic Acid | 0.0071 | 0.0713 | 0.6514 | 0.8965 | 0.7964 | 0.9364 |
| †*L*-Theanine | 0.7710 | 0.9279 | 0.9722 | 0.9951 | 0.5038 | 0.8138 |
| Malic acid | 0.0805 | 0.4264 | 0.7532 | 0.9266 | 0.7021 | 0.9091 |
| Malitol | 0.0462 | 0.3163 | 0.6845 | 0.9091 | 0.5582 | 0.8372 |
| Mannitol | 0.0339 | 0.2548 | 0.7568 | 0.9279 | 0.0205 | 0.1785 |
| Mannose | 0.1441 | 0.5267 | 0.6414 | 0.8918 | 0.5981 | 0.8590 |
| Methylglutaric acid | 0.2133 | 0.5921 | 0.2677 | 0.6409 | 0.1606 | 0.5404 |
| Methyl-phenylacetate | 0.3935 | 0.7358 | 0.3734 | 0.7226 | 0.4361 | 0.7609 |
| Mytilitol | 0.1968 | 0.5791 | 0.8818 | 0.9661 | 0.7392 | 0.9206 |
| *N*-Acetylaspartate | 0.0709 | 0.3969 | 0.4212 | 0.7529 | 0.8255 | 0.9524 |
| *N*-Acetylglutamate | 0.1556 | 0.5380 | 0.5246 | 0.8224 | 0.7221 | 0.9112 |
| *N*-Acetyl-L-alanine | 0.0000 | 0.0000 | 0.6357 | 0.8880 | 0.4384 | 0.7609 |
| ††*N*-Acetyl-*L*-methionine | 0.1207 | 0.5026 | 0.8282 | 0.9524 | 0.9550 | 0.9895 |
| *N*-Acetylneuraminic acid | 0.0877 | 0.4351 | 0.5879 | 0.8505 | 0.9777 | 0.9953 |
| Nonate | 0.7126 | 0.9112 | 0.9713 | 0.9951 | 0.4746 | 0.7919 |
| Oxalic acid | 0.1452 | 0.5267 | 0.2018 | 0.5838 | 0.7178 | 0.9112 |
| Pantoic acid | 0.0702 | 0.3969 | 0.5141 | 0.8195 | 0.2907 | 0.6526 |
| Pantothenic acid | 0.1848 | 0.5641 | 0.5832 | 0.8477 | 0.9674 | 0.9948 |
| *p*-Hydroxyphenylacetic acid | 0.4294 | 0.7573 | 0.9980 | 0.9980 | 0.7080 | 0.9091 |
| Phytanic acid | 0.3874 | 0.7314 | 0.4302 | 0.7573 | 0.9824 | 0.9955 |
| ††Propionylcarnitine | 0.0000 | 0.0021 | 0.7511 | 0.9260 | 0.8479 | 0.9613 |
| Protocatechuic acid | 0.6726 | 0.9002 | 0.1805 | 0.5620 | 0.6657 | 0.8994 |
| Pyroglutamic acid | 0.0678 | 0.3969 | 0.5688 | 0.8417 | 0.9089 | 0.9780 |
| Pyruvic acid | 0.1231 | 0.5026 | 0.9390 | 0.9895 | 0.4776 | 0.7948 |
| Quinic acid | 0.0661 | 0.3909 | 0.2490 | 0.6318 | 0.1203 | 0.5026 |
| Quinolinic acid | 0.0621 | 0.3784 | 0.8288 | 0.9524 | 0.9860 | 0.9957 |
| Raffinose | 0.2532 | 0.6347 | 0.5104 | 0.8179 | 0.2396 | 0.6179 |
| Ribulose | 0.0208 | 0.1786 | 0.8579 | 0.9613 | 0.8721 | 0.9657 |
| Sorbitol | 0.1034 | 0.4699 | 0.9797 | 0.9953 | 0.7022 | 0.9091 |
| †Stearic acid | 0.9491 | 0.9895 | 0.5524 | 0.8306 | 0.4228 | 0.7529 |
| Succinic acid | 0.0844 | 0.4290 | 0.3333 | 0.6897 | 0.4654 | 0.7808 |
| Tartaric acid | 0.2395 | 0.6179 | 0.5391 | 0.8246 | 0.5927 | 0.8534 |
| Taurine | 0.0836 | 0.4290 | 0.6949 | 0.9091 | 0.9768 | 0.9953 |
| ††Theophylline | 0.1635 | 0.5441 | 0.3218 | 0.6875 | 0.8684 | 0.9651 |
| †Tryptamine | 0.0705 | 0.3969 | 0.4894 | 0.8012 | 0.7903 | 0.9354 |
| Uridine | 0.0757 | 0.4079 | 0.2696 | 0.6413 | 0.4051 | 0.7466 |
| ††Vanillin | 0.1123 | 0.4954 | 0.6019 | 0.8605 | 0.3605 | 0.7014 |
| Xylulose | 0.1147 | 0.5025 | 0.9134 | 0.9804 | 0.8737 | 0.9657 |

**Three-way ANOVA reveals B vitamin-dependent changes and their association with brain atrophy**

**SI Table 8** List of NMR identified metabolites and results of three-way ANOVA (main class effects).

| Metabolite | Bin  (ppm) | Class 1 | | Class 2 | | Class 3 | |
| --- | --- | --- | --- | --- | --- | --- | --- |
|  |  | *p*-value | FDR | *p*-value | FDR | *p*-value | FDR |
| High density lipoproteins | [0.85..0.87] | 0.43 | 0.82 | 0.18 | 0.70 | 0.92 | 0.95 |
| Very low-density lipoproteins | [0.87..0.89] | 0.22 | 0.75 | 0.25 | 0.76 | 0.35 | 0.77 |
| Isoleucine | [0.95..0.97] | 0.52 | 0.85 | 0.09 | 0.57 | 0.71 | 0.89 |
| Leucine | [0.97..0.99] | 0.14 | 0.66 | 0.20 | 0.74 | 0.49 | 0.82 |
| Hydroxybutyrate | [1.17..1.19] | 0.75 | 0.89 | 0.04 | 0.40 | 0.47 | 0.82 |
| Low density lipoproteins | [1.23..1.25] | 0.75 | 0.89 | 0.39 | 0.79 | 0.33 | 0.77 |
| Low/Very-low density lipoproteins | [1.29..1.31] | 0.28 | 0.77 | 0.27 | 0.77 | 0.36 | 0.77 |
| Alanine | [1.47..1.49] | 0.13 | 0.66 | 0.36 | 0.77 | 0.11 | 0.59 |
| βCH_2_ | [1.59..1.61] | 0.49 | 0.83 | 0.57 | 0.86 | 0.43 | 0.82 |
| /=CH-CH_2_-CH= | [1.99..2.01] | 0.68 | 0.89 | 0.14 | 0.66 | 0.58 | 0.86 |
| Proline | [2.01..2.03] | 0.35 | 0.77 | 0.17 | 0.70 | 0.63 | 0.89 |
| *N*-acetyl glycoprotein A (NAC1) | [2.03..2.05] | 0.53 | 0.85 | 0.30 | 0.77 | 0.87 | 0.92 |
| *N*-acetyl glycoprotein B (NAC2) | [2.07..2.09] | 0.07 | 0.54 | 0.89 | 0.93 | 0.75 | 0.89 |
| Glutamine | [2.13..2.15] | 0.04 | 0.40 | 0.70 | 0.89 | 0.57 | 0.86 |
| Acetoacetate | [2.21..2.23] | 0.45 | 0.82 | 0.67 | 0.89 | 0.03 | 0.40 |
| Valine/Proline | [2.29..2.31] | 0.53 | 0.85 | 0.60 | 0.87 | 0.70 | 0.89 |
| Glutamate | [2.35..2.37] | 0.37 | 0.77 | 0.75 | 0.89 | 0.22 | 0.75 |
| Creatinine | [3.03..3.05] | 0.22 | 0.75 | 0.86 | 0.92 | 0.14 | 0.66 |
| Mobile -N(CH_3_)_3_/ | [3.19..3.21] | 0.44 | 0.82 | 0.63 | 0.89 | 0.48 | 0.82 |
| mobile -N(CH_3_)_3_/free choline | [3.21..3.23] | 0.10 | 0.57 | 0.86 | 0.92 | 0.71 | 0.89 |
| Histidine | [3.31..3.33] | 0.10 | 0.57 | 0.65 | 0.89 | 0.17 | 0.70 |
| Glucose | [3.55..3.57] | 0.48 | 0.82 | 0.37 | 0.77 | 0.70 | 0.89 |
| Threonine | [3.59..3.61] | 0.48 | 0.82 | 0.64 | 0.89 | 0.76 | 0.89 |
| Myoinositol | [3.61..3.63] | 0.21 | 0.75 | 0.76 | 0.89 | 0.13 | 0.66 |
| Lactate | [4.11..4.13] | 0.46 | 0.82 | 0.41 | 0.80 | 0.94 | 0.96 |
| Unsaturated lipid | [5.30..5.32] | 0.08 | 0.54 | 0.23 | 0.75 | 0.52 | 0.85 |
| Tyrosine | [6.88..6.90] | 0.28 | 0.77 | 0.99 | 0.99 | 0.04 | 0.40 |
| Phenylalanine | [7.56..7.58] | 0.11 | 0.61 | 0.63 | 0.89 | 0.23 | 0.75 |

**Two-Way ANOVA *Post hoc* Results**

A Two-Way ANOVA with Tukey's *post hoc* analysis of follow-up metabolite levels, adjusted for baseline, revealed that treatment (B vitamin administration) is the primary factor driving interclass differences; however, secondary effects associated with brain atrophy progression were also observed.

**SI Table 9** Two-Way ANOVA *post hoc* results for the metabolites selected in the three-way ANOVA analysis. The follow-up levels adjusted for the baseline levels were used for each metabolite.

| Metabolite | Combined class 1 | Combined class 2 | *p* value |
| --- | --- | --- | --- |
| 2-Ketoglutaric acid | placebo stable | placebo progressor | 0.4084 |
| 2-Ketoglutaric acid | placebo stable | vitamin stable | 0.2008 |
| 2-Ketoglutaric acid | placebo stable | vitamin progressor | 0.6047 |
| 2-Ketoglutaric acid | placebo progressor | vitamin stable | 0.0192 |
| 2-Ketoglutaric acid | placebo progressor | vitamin progressor | 0.2199 |
| 2-Ketoglutaric acid | vitamin stable | vitamin progressor | 0.6183 |
| Glutamic acid | placebo progressor | placebo stable | 0.3476 |
| Glutamic acid | placebo progressor | vitamin stable | 0.0331 |
| Glutamic acid | placebo progressor | vitamin progressor | 0.1358 |
| Glutamic acid | placebo stable | vitamin stable | 0.4737 |
| Glutamic acid | placebo stable | vitamin progressor | 0.6458 |
| Glutamic acid | vitamin stable | vitamin progressor | 0.9612 |
| Succinic acid | placebo progressor | placebo stable | 0.2652 |
| Succinic acid | placebo progressor | vitamin stable | 0.0097 |
| Succinic acid | placebo progressor | vitamin progressor | 0.3804 |
| Succinic acid | placebo stable | vitamin stable | 0.1271 |
| Succinic acid | placebo stable | vitamin progressor | 0.9920 |
| Succinic acid | vitamin stable | vitamin progressor | 0.2043 |
| Glucose | placebo progressor | placebo stable | 0.0358 |
| Glucose | placebo progressor | vitamin stable | 0.0492 |
| Glucose | placebo progressor | vitamin progressor | 0.1621 |
| Glucose | placebo stable | vitamin stable | 0.5187 |
| Glucose | placebo stable | vitamin progressor | 0.6838 |
| Glucose | vitamin stable | vitamin progressor | 0.8848 |
| Malic acid | placebo progressor | vitamin stable | 0.0403 |
| Malic acid | placebo progressor | placebo stable | 0.4995 |
| Malic acid | placebo progressor | vitamin progressor | 0.2112 |
| Malic acid | vitamin stable | placebo stable | 0.1750 |
| Malic acid | vitamin stable | vitamin progressor | 0.7855 |
| Malic acid | placebo stable | vitamin progressor | 0.4239 |
| *N*-Acetylglutamate | placebo progressor | vitamin stable | 0.2045 |
| *N*-Acetylglutamate | placebo progressor | placebo stable | 0.3778 |
| *N*-Acetylglutamate | placebo progressor | vitamin progressor | 0.3011 |
| *N*-Acetylglutamate | vitamin stable | placebo stable | 0.6831 |
| *N*-Acetylglutamate | vitamin stable | vitamin progressor | 0.9842 |
| *N*-Acetylglutamate | placebo stable | vitamin progressor | 0.6848 |
| Glutaric acid | placebo progressor | placebo stable | 0.8056 |
| Glutaric acid | placebo progressor | vitamin stable | 0.0727 |
| Glutaric acid | placebo progressor | vitamin progressor | 0.9891 |
| Glutaric acid | placebo stable | vitamin stable | 0.1380 |
| Glutaric acid | placebo stable | vitamin progressor | 0.8504 |
| Glutaric acid | vitamin stable | vitamin progressor | 0.1390 |
| Lactic acid | placebo progressor | placebo stable | 0.4993 |
| Lactic acid | placebo progressor | vitamin stable | 0.0670 |
| Lactic acid | placebo progressor | vitamin progressor | 0.3424 |
| Lactic acid | placebo stable | vitamin stable | 0.1897 |
| Lactic acid | placebo stable | vitamin progressor | 0.5384 |
| Lactic acid | vitamin stable | vitamin progressor | 0.6871 |
| Pyruvic acid | vitamin stable | placebo stable | 0.3635 |
| Pyruvic acid | vitamin stable | placebo progressor | 0.2423 |
| Pyruvic acid | vitamin stable | vitamin progressor | 0.9134 |
| Pyruvic acid | placebo stable | placebo progressor | 0.6954 |
| Pyruvic acid | placebo stable | vitamin progressor | 0.5330 |
| Pyruvic acid | placebo progressor | vitamin progressor | 0.4765 |
| 2-Ketobutyric acid | placebo stable | placebo progressor | 0.3326 |
| 2-Ketobutyric acid | placebo stable | vitamin stable | 0.1647 |
| 2-Ketobutyric acid | placebo stable | vitamin progressor | 0.8150 |
| 2-Ketobutyric acid | placebo progressor | vitamin stable | 0.0300 |
| 2-Ketobutyric acid | placebo progressor | vitamin progressor | 0.3534 |
| 2-Ketobutyric acid | vitamin stable | vitamin progressor | 0.3477 |
| Quinolinic acid | placebo stable | placebo progressor | 0.3338 |
| Quinolinic acid | placebo stable | vitamin stable | 0.1245 |
| Quinolinic acid | placebo stable | vitamin progressor | 0.9387 |
| Quinolinic acid | placebo progressor | vitamin stable | 0.0091 |
| Quinolinic acid | placebo progressor | vitamin progressor | 0.3376 |
| Quinolinic acid | vitamin stable | vitamin progressor | 0.3020 |
| Glutamine | vitamin stable | placebo stable | 0.0232 |
| Glutamine | vitamin stable | placebo progressor | 0.0452 |
| Glutamine | vitamin stable | vitamin progressor | 0.0476 |
| Glutamine | placebo stable | placebo progressor | 0.4978 |
| Glutamine | placebo stable | vitamin progressor | 0.7058 |
| Glutamine | placebo progressor | vitamin progressor | 0.4270 |

**The metabolomic differences are primarily associated with B vitamins rather than brain atrophy**

***
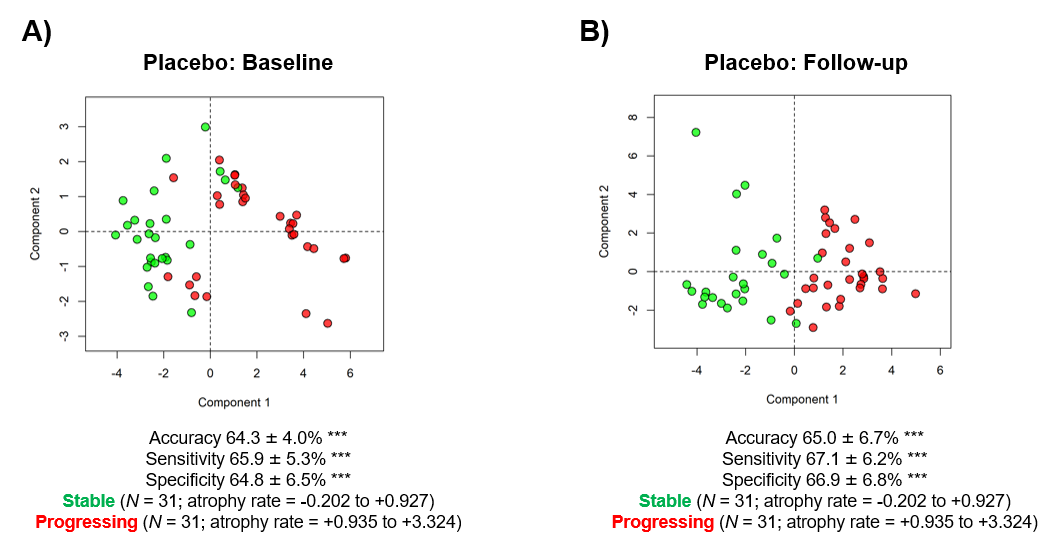
***

**SI Fig. 9** OPLS-DA scores plot showing the separation between stable placebo controls (green; *N* = 31; atrophy rate: -0.202 to +0.927) and progressing (worsening) placebo controls (red; *N* = 31; atrophy rate: +0.935 to +3.324) stratified based on the rate of brain atrophy progression. a) baseline samples; b) follow-up samples. The minimal differences in separation suggest that brain atrophy progression has only a limited impact on the metabolome. Consequently, the differences observed in the models presented in the main text (Fig. 1) are predominantly reflective of the treatment effect.

**Pearson correlation analysis for B vitamin treated-individuals with slow rate of brain atrophy progression (“responders to treatment”)**


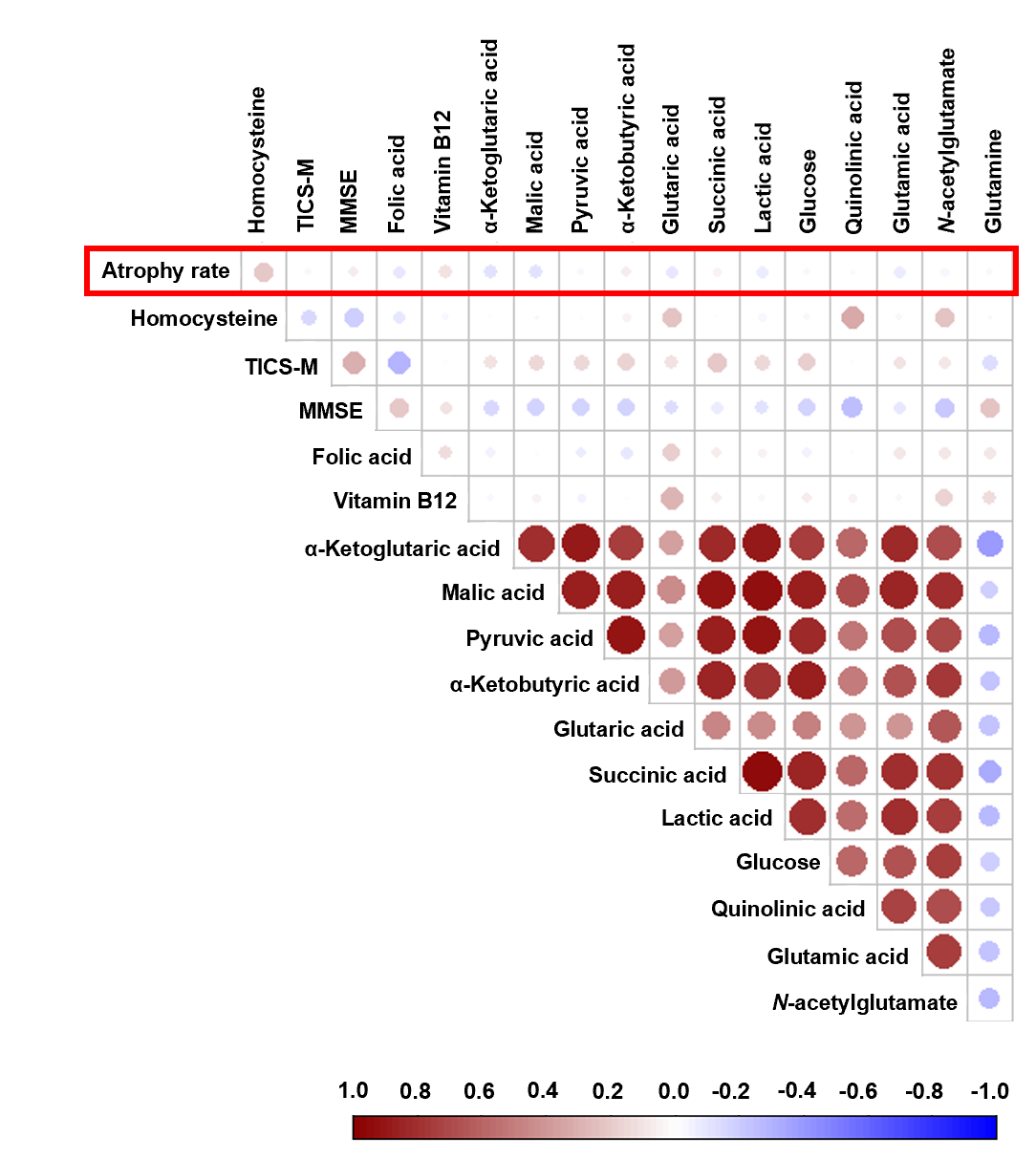


**SI Fig. 10** Pearson correlation analysis of significantly altered metabolites, homocysteine (total homocysteine), folate, and vitamin B12 (all follow-up levels), atrophy rate (highlighted in the red box), and cognitive test scores (MMSE and TICS-M). The discrepancy between TICS-M and MMSE may be attributed to the higher sensitivity of TICS-M, particularly in mild cognitive impairment (MCI). Data include all compliant individuals with available MRI scans at the follow-up time point who received B vitamins and were characterised by a slow rate of brain atrophy ("stable"; *N* = 51).

**Metabolic pathway analysis reveals B vitamins effects on amino acid and central carbon metabolism**

**SI Table 10** Results of the metabolic pathway analysis comparing B vitamin-treated individuals and placebo controls, including pathway significance and impact.

| Pathway | Total Compounds | Total  Hits | Pathway p-value | Pathway impact |
| --- | --- | --- | --- | --- |
| Biosynthesis of unsaturated fatty acids | 36 | 2 | 0.0057 | 0.0000 |
| Nicotinate and nicotinamide metabolism | 15 | 2 | 0.0073 | 0.0000 |
| Fatty acid degradation | 33 | 3 | 0.0201 | 0.0000 |
| Glycine, serine and threonine metabolism | 21 | 1 | 0.0228 | 0.0000 |
| Pentose metabolism | 19 | 4 | 0.0313 | 0.2410 |
| Lipoic acid metabolism | 28 | 2 | 0.0361 | 0.0068 |
| Alanine, aspartate and glutamate metabolism | 28 | 6 | 0.0464 | 0.5072 |
| Histidine metabolism | 16 | 2 | 0.0578 | 0.0000 |
| Glycolysis or Gluconeogenesis | 26 | 3 | 0.0644 | 0.0442 |
| Glyoxylate and dicarboxylate metabolism | 32 | 6 | 0.0683 | 0.1400 |
| Citrate cycle (TCA cycle) | 20 | 5 | 0.0811 | 0.2586 |
| Arginine biosynthesis | 14 | 3 | 0.0830 | 0.1223 |
| Galactose metabolism | 27 | 7 | 0.1119 | 0.1871 |
| Tyrosine metabolism | 42 | 1 | 0.1477 | 0.0000 |
| Pentose and glucuronate interconversions | 19 | 4 | 0.1477 | 0.0000 |
| Pyruvate metabolism | 23 | 3 | 0.2028 | 0.2197 |
| Arginine and proline metabolism | 36 | 2 | 0.2671 | 0.0000 |
| Glycerolipid metabolism | 16 | 1 | 0.2706 | 0.0935 |
| Pantothenate and CoA biosynthesis | 20 | 3 | 0.3443 | 0.0979 |
| Cysteine and methionine metabolism | 33 | 2 | 0.3600 | 0.1785 |
| Glutathione metabolism | 28 | 2 | 0.3737 | 0.0268 |
| Ascorbate and aldarate metabolism | 9 | 1 | 0.4288 | 0.5238 |
| Inositol phosphate metabolism | 30 | 1 | 0.4288 | 0.0000 |
| Tryptophan metabolism | 41 | 1 | 0.4481 | 0.0139 |
| Valine, leucine and isoleucine degradation | 40 | 1 | 0.5563 | 0.0108 |
| Valine, leucine and isoleucine biosynthesis | 8 | 1 | 0.5563 | 0.0000 |
| Fructose and mannose metabolism | 20 | 3 | 0.6080 | 0.1308 |
| Propanoate metabolism | 22 | 1 | 0.6337 | 0.0000 |
| Neomycin, kanamycin and gentamicin biosynthesis | 2 | 1 | 0.6561 | 0.0000 |
| Amino sugar and nucleotide sugar metabolism | 42 | 3 | 0.6949 | 0.0637 |
| One carbon pool by folate | 26 | 1 | 0.7269 | 0.0449 |
| Purine metabolism | 70 | 1 | 0.7454 | 0.0000 |
| Pyrimidine metabolism | 39 | 1 | 0.8367 | 0.0399 |
| Starch and sucrose metabolism | 18 | 3 | 0.8779 | 0.5601 |
| Butanoate metabolism | 15 | 2 | 0.8794 | 0.0000 |
| Taurine and hypotaurine metabolism | 8 | 1 | 0.9250 | 0.4286 |
| Primary bile acid biosynthesis | 46 | 1 | 0.9250 | 0.0076 |
| Porphyrin metabolism | 31 | 1 | 0.9398 | 0.0000 |
| Nitrogen metabolism | 6 | 1 | 0.9398 | 0.0000 |
